# Cannabidiolic Acid Rescues Deficits in Hippocampal Long-Term Potentiation in Models of Alzheimer’s Disease: An Electrophysiological and Proteomic Analysis

**DOI:** 10.3390/ijms26104944

**Published:** 2025-05-21

**Authors:** Beatriz Gil, Mairéad Sullivan, Caitriona Scaife, Jeffrey C. Glennon, Caroline Herron

**Affiliations:** 1 School of Biomolecular and Biomedical Sciences, University College Dublin, Conway Institute, Dublin 4, Ireland; beagilla@gmail.com; 2 School of Medicine and Conway Institute of Biomolecular and Biomedical Research, University College Dublin, Conway Institute, Dublin 4, Ireland; maireadsullivan33@gmail.com (M.S.); jeffrey.glennon@ucd.ie (J.C.G.); 3 Conway Institute, University College, Dublin 4, Ireland; caitriona.scaife@ucd.ie

**Keywords:** Alzheimer’s disease, CBDA, long-term potentiation, proteomics, synaptic plasticity, APP/PS1, electrophysiology

## Abstract

In this study, we have examined the neuroprotective effects of cannabidiolic acid (CBDA) in models of Alzheimer’s disease (AD). We used in vitro electrophysiological recording in hippocampal slices and performed proteomic analysis of cortical tissue from APP_swe_/PS1dE9 (APP/PS1) mice. In wild-type (WT) slices from C57BL6 mice, acute treatment with CBDA (10 μM) did not alter levels of hippocampal long-term potentiation (LTP); however, it did reverse the attenuation of LTP produced by acute beta amyloid peptide (Aβ_42_). We also examined the effects of CBDA or vehicle in APP/PS1 mice and WT littermates over a 5-week period at 8 months. LTP levels recorded in slices from WT mice treated with CBDA at 1, 10, or 30 mg/kg (IP) or vehicle were similar. LTP was attenuated in slices from vehicle-treated APP/PS1 compared to vehicle-treated WT mice, while treatment of APP/PS1 mice with all doses of CBDA reversed the deficits in LTP. There was also a deficit in paired-pulse facilitation (PPF) in vehicle-treated APP/PS1 compared to WT, indicating altered synaptic function and transmitter release; this was reversed in slices from CBDA-treated APP/PS1 mice. Levels of cortical soluble Aβ_42_ were similar across CBDA- and vehicle-treated groups; however, the level of aggregated Aβ_42_ was decreased in the CBDA-treated group. Proteomic analysis of cortical tissue from APP/PS1 cortex compared to WT revealed alterations in protein expression, with pathway enrichment analyses suggesting implicated canonical pathways, including mitochondrial dysfunction, protein sorting, and synaptogenesis; all were significantly improved by CBDA treatment. These changes likely facilitate the improvement in synaptic transmission and LTP we observed following CBDA treatment in APP/PS1 mice. This research suggests that CBDA should be considered a novel therapy for AD.

## 1. Introduction

Alzheimer’s disease (AD) causes progressive neuronal death and a terminal decline in cognitive function. The pathological hallmarks of AD are the deposition of extracellular plaques comprised of amyloid beta (Aβ) peptide and intracellular neurofibrillary tangles (NFTs) composed of hyperphosphorylated tau (τ) protein. Early-onset or familial AD is usually linked to mutations in the genes encoding amyloid precursor protein (APP), presenilin 1 (PSEN1), and presenilin 2 (PSEN2), all of which increase levels of neurotoxic Aβ_42_. Sporadic AD is also associated with the APOE4 allele known to decrease clearance of Aβ [1]. The amyloid cascade hypothesis [2] proposed that Aβ is the main trigger for AD pathology and neuronal dysfunction. A consequence of the decreased clearance and/or increased production of Aβ_42_ is the formation of toxic soluble oligomers, fibrils, and, ultimately, Aβ plaques [3,4], which can activate proinflammatory processes [5]. More recently, other mechanisms, including mitochondrial dysfunction, have been implicated as a consequence of AD pathology [6,7] The mitochondrial cascade hypothesis posits that mitochondrial dysfunction drives the pathogenesis of AD, as baseline mitochondrial function and mitochondrial change rates influence the progression of cognitive decline. However, this has not yet been fully delineated in AD mouse models where examination of both synaptic and mitochondrial markers is required next to functional electrophysiological metrics of synaptic plasticity. The expression of both synaptic and mitochondrial proteins can be assessed using mass spectrometry proteomic approaches.

Hippocampal long-term potentiation (LTP) is a form of synaptic plasticity thought to be linked to memory. Soluble Aβ oligomers depress LTP [8,9,10,11]. LTP has also been used to examine agents that could potentially protect neuronal plasticity against toxic Aβ [12,13]. While the physiological role of Aβ is uncertain, low levels are thought to be required for cGMP-induced LTP [14].

While the increased incidence of AD due to longevity imposes an increasing socio-economic burden, the therapeutic agents currently available do not alter disease progression. Recent antibody therapies offer hope for the provision of treatments that may reduce AD progress [15]; however, there is still a need to develop affordable therapies for AD.

We have previously reported that the non-psychoactive component of *Cannabis sativa*, cannabidiol (CBD), provided neuroprotection against Aβ_42_-mediated inhibition of LTP in an acute in vitro model of AD [11]; a potential mechanism of action is via peroxisome proliferator active receptor gamma (PPARγ). CBD is known to act as an antioxidant [16,17] and also as an anti-inflammatory and neuroprotective agent [18] against Aβ. While *Cannabis sativa* contains over 120 cannabinoid-type compounds, we have focussed our attention on cannabidiolic acid (CBDA), which is the precursor to CBD. CBDA is one of the most abundant phytocannabinoids in fibre-type plants and seed-oil hemp varieties.

CBDA application in animal models has been shown to have anti-hyperalgesia [19,20], anti-convulsant [21], anti-inflammatory [22], and anti-obesity properties [23]. CBDA is also a powerful antioxidant [24]. It has also been identified as an inhibitor of cyclo-oxygenase 2 [25] and has recently been shown to protect against the loss of learning and memory caused by the application of Aβ in vivo [26]. As CBDA is the chemical precursor to CBD [21,27,28,29], some of the therapeutic properties could be due its metabolite CBD, as mentioned previously.

There is currently no literature available on the impact of CBDA on hippocampal LTP or its effects against Aβ-mediated impairments in LTP. In the current study, using hippocampal slices (C57B6 mice), we investigated the effects of acute CBDA application in vitro against attenuation of LTP produced by acute application of oligomeric Aβ_42_. In addition, we further investigated the effects of CBDA in the APPswe/PS1dE9 (APP/PS1) mouse model of AD [30]. These mice overexpress the human APP-encoding gene with the Swedish mutation and the delta E9 mutation in presenilin. Consequently, they show an age-dependent increase in Aβ, producing cognitive deficits at 7 months associated with deposition of Aβ plaques [31]. We have previously shown deficits in LTP in hippocampal slices prepared from this AD mouse model [13].

The current study examines LTP and synaptic transmission in hippocampal slices from control wild-type C57B6 mice and from CBDA- and vehicle-treated APP/PS1 mice. Levels of Aβ_40_ and Aβ_42_ in cortical tissue were also measured using ELISA. Mass spectrometry-based proteomic analysis was also performed on cortical tissue, followed by protein enrichment analysis to assess the potential signalling and metabolic pathways that may be altered between our WT and APP/PS1 mice treated with vehicle or CBDA. We found that acute CBDA reversed Aβ-mediated attenuation of LTP. In addition, systemic treatment with CBDA rescued the deficit in LTP and paired-pulse facilitation observed in hippocampal slices from vehicle-treated APP/PS1 mice. Proteomic analysis revealed deficits in mitochondrial function and synaptogenesis, among other neuronal signalling pathways in APP/PS1 cortical tissue, which were reversed following treatment with CBDA.

## 2. Results

### 2.1. LTP in Hippocampal Slices from C57B6 Mice

**Figure 1:**
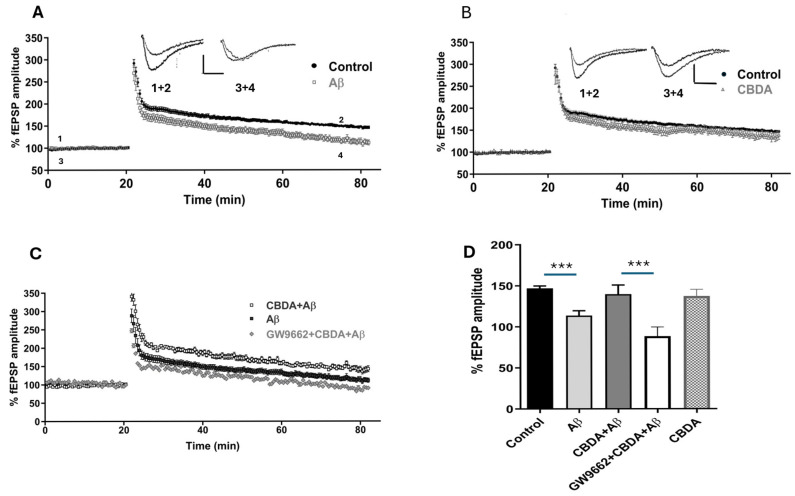
CBDA restores Aβ-induced LTP impairment. Graphs and bar charts showing the level of LTP under different conditions. (**A**) LTP recorded in the presence of Aβ (113.6 ± 5.9, *n* = 20) was significantly reduced compared to the control (146.9 ± 2.9, *n* = 62) (*** denotes *p* < 0.001). Scale bar: 0.5 mV/10 ms. (**B**) The magnitude of LTP in the presence of CBDA (10 μM; 137.3 ± 8.2, *n* = 8) was similar to that of the control (146.9 ± 2.9, *n* = 62). (**C**) LTP graph showing that LTP recorded in CBDA+Aβ was similar to that of the control. The effect of CBDA on Aβ-mediated LTP was significantly reversed by the PPARγ antagonist GW9662 (control: 146.9 ± 2.9, *n* = 62; Aβ: 113.6 ± 5.9, *n* = 20; CBDA+ Aβ: 139.6 ± 11.2, *n* = 8; GW9662: 88.41 ± 11.2, *n* = 6; *p* < 0.001. (**D**) Bar chart showing LTP levels recorded in all conditions. Data set analysed using one-way ANOVA Bonferroni multiple-comparisons test, bars represent SEM.

When slices were perfused with Aβ (amyloid-derived diffusible ligands: Aβ_42_; 500 nM) for 30 min prior to LTP induction, LTP was significantly reduced compared to control (Figure 1A, *p* < 0.001). The levels of LTP recorded in the presence of CBDA (10 μM) and in vehicle-control conditions were similar (Figure 1B) (*p* > 0.05). When CBDA was applied to slices 30 min prior to Aβ, however, the level of LTP was similar to that of control conditions (Figure 1C, *p* > 0.05). PTP and PPF were unaltered by the combination of CBDA+Aβ compared to either the control or the Aβ-treated group. Previously, we found that the neuroprotection offered by acute CBD could be attenuated by inhibition of PPARγ [11]. We therefore examined the effects of addition of GW9662 (2 μM) and CBDA prior to Aβ on LTP. We found that under those conditions the partial neuroprotective effects of CBDA were reduced (Figure 1C,D, bar chart).

### 2.2. LTP in Hippocampal Slices from APP/PS1 Mice and Wild-Type Littermates Chronically Treated with CBDA or Vehicle

**Figure 2:**
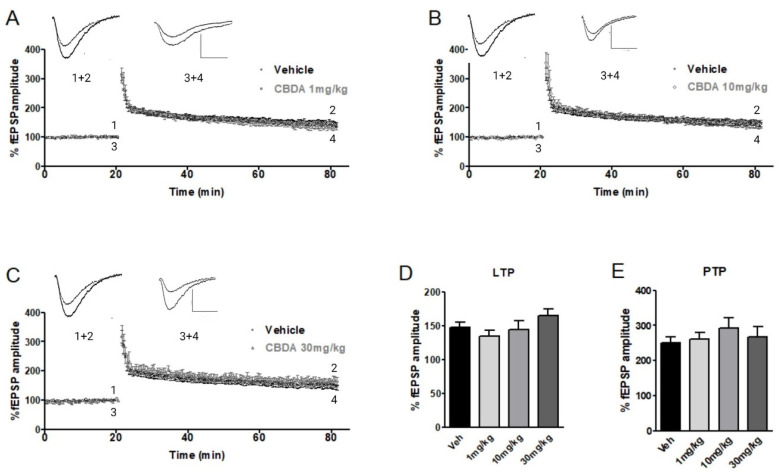
Effects of chronic CBDA treatment on LTP in 9-month-old wild-type mice. Treatment with CBDA (1, 10, or 30 mg/kg) did not significantly alter LTP amplitude in hippocampal slices. Sample traces represent fEPSP at 15–20 min of baseline (**1**, **3**) and LTP 55–60 min after HFS (**2**, **4**) in wild-type mice treated with CBDA or vehicle. Scale bar: 0.5 mV /10 ms. (**A**) Following CBDA 1 mg/kg, LTP measured 134.8 ± 8.28 (*n* = 11). (**B**) CBDA 10 mg/kg, LTP measured 144.6 ± 13.2.,9 (*n* = 7). (**C**) CBDA at 30 mg/kg; LTP measured 165.8 ± 9.45 (*n* = 6). (**D**) Bar chart showing no significant difference in LTP levels comparing vehicle-treated wild-type mice (147.9 ± 8.01, *n* = 11) or mice treated with 1, 10, or 30 mg/kg CBDA. (**E**) Levels of PTP were also similar across all groups (vehicle: 251.2 ± 16.0, *n* = 11; 1 mg/kg: 260.0 ± 19.2, *n* = 11; 10 mg/kg: 292.7 ± 28.3, *n* = 7; 30 mg/kg: 266.1 ± 29.7, *n* = 6). Data presented as mean ± SEM and analysed using one-way ANOVA (and Bonferroni’s post hoc test to compare all pairs of columns).

Due to the results obtained with acute applications of CBDA and Aβ, we investigated the effects of CBDA on the APP/PS1 mouse model of AD. The aim of our next series of experiments was to examine LTP in hippocampal slices from APP/PS1 mice and their WT littermates following a 5-week period of i.p. injections with CBDA (1, 10 or 30 mg/kg) or vehicle. LTP levels in hippocampal slices from C57BL6 (wild-type littermates) were compared between groups treated with either vehicle or CBDA at 1, 10, or 30 mg/kg. Wild-type (WT) mice receiving vehicle exhibited LTP values that were used as the control level (147.9 ± 8.0; *n* = 11). While there was some variation in the level of LTP recorded in slices from WT mice treated with CBDA (1 mg/kg: 134.8 ± 8.2; *n* = 11, Figure 2A; 10 mg/kg: 144.6 ± 13.2; *n* = 7, Figure 2B; and 30 mg/kg: 165.8 ± 9.4; *n* = 6, Figure 2C), there was no statistical difference in LTP between groups (see Figure 2D, bar chart). In addition, PTP did not differ between treatment groups (vehicle: 251.2 ± 16.0, *n* = 11; 1 mg/kg: 260.0 ± 19.2, *n* = 11; 10 mg/kg: 292.7 ± 28.3, *n* = 7; 30 mg/kg: 266.1 ± 29.7, *n* = 6). Our data suggest that chronic treatment with CBDA alone does not significantly alter short-term plasticity or LTP.

### 2.3. LTP in Vehicle-Treated and CBDA-Treated APP/PS1 Mice

**Figure 3:**
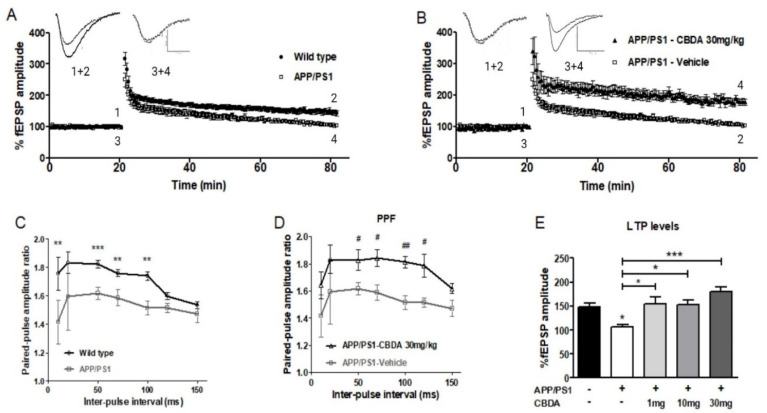
Chronic treatment with CBDA restores impaired levels of LTP in slices from 9-month-old APP/PS1 mice. (**A**) LTP recorded in APP/PS1 mice (3 + 4) and wild-type littermates (1 + 2); sample data traces: scale bar 0.5 mV /10 ms. (**B**) Graph of LTP recorded in slices from APP/PS1 mice treated with 30 mg/kg CBDA (3 + 4) and vehicle (1 + 2). (**C**) Paired-pulse facilitation (PPF) ratio was significantly decreased in slices from vehicle-treated APP/PS1 mice (*n* = 6) compared to wild-type littermates (*n* = 9) across a range of inter-pulse intervals (** *p* < 0.01; *** *p* < 0.001, unpaired t-test). (**D**) This deficit was significantly restored following chronic treatment with 30 mg/kg CBDA (*n* = 5) (unpaired *t*-test; # *p* < 0.05, ## *p* < 0.01). (**E**) Bar chart showing that LTP levels recorded in slices from vehicle-treated APP/PS1 mice (107.1 ± 4.3, *n* = 6) were significantly lower than in vehicle-treated wild-type mice (147.9 ± 8.0, *n* = 11). LTP was significantly increased in APP/PS1 groups following CBDA treatment with 1 mg/kg (155.1 ± 14.6, *n* = 6) (* *p* ≤ 0.5), 10 mg/kg (153.2 ± 9.9, *n* = 8) (* *p* ≤ 0.5), and 30 mg/kg CBDA (180.5 ± 8.8, *n* = 5) (*** *p* ≤ 0.001). Data presented as mean ± SEM and analysed using one-way ANOVA (and Bonferroni’s post hoc test to compare all pairs of columns).

We found that there was a significant deficit in LTP levels in slices from vehicle-treated APP/PS1 mice (107.1 ± 4.3; *n* = 6) compared to WT littermates (147.9 ± 8.0; *n* = 11) (Figure 3A). These results were similar to our previous observations using this mouse model [13]. In addition, when we examined levels of PPF at inter-pulse intervals of 10 to 150 ms in slices from vehicle-treated APP/PS1 mice, we found that PPF was depressed compared to levels recorded in slices from vehicle-treated WT littermates (see Figure 3C, *p* < 0.05).

LTP was recorded in hippocampal slices from APP/PS1 mice treated with either vehicle or CBDA at 1, 10, or 30 mg/kg. Chronic treatment with CBDA during a 5-week period significantly increased levels of LTP in slices from APP/PS1 mice (1 mg/kg: 155.1 ± 14.5, *p* < 0.01; *n* = 6), (10 mg/kg: 153.2 ± 9.9, *p* < 0.05, *n* = 8), (30 mg/kg: 180.5 ± 8.8; *p* < 0.001; *n* = 5) compared to vehicle-treated APP/PS1 (107.1 ± 4.3; *n* = 6) (Figure 3E). Paired-pulse facilitation (PPF) was examined in all treatment groups. We found that in slices from APP/PS1 mice treated with CBDA (30 mg/kg) there was a higher paired-pulse ratio, similar to values recorded from control vehicle-treated WT mice (*p* < 0.05, Figure 3D).

### 2.4. Levels of Beta Amyloid Peptide (Aβ)

**Figure 4:**
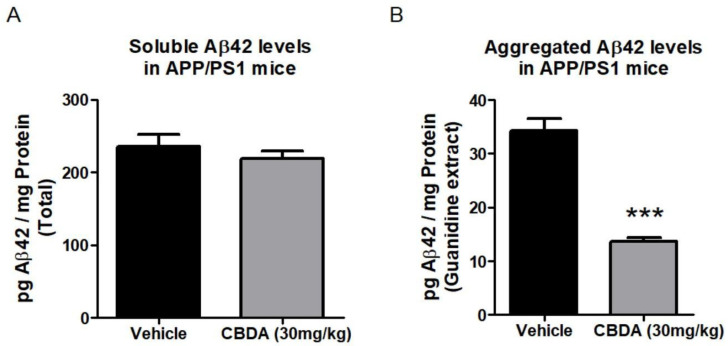
Effect of chronic treatment with CBDA (30 mg/kg) on the levels of soluble and aggregated extracts of Aβ_42_ in APP/PS1 mice at 9 months. (**A**) Bar chart showing no significant changes in levels of soluble Aβ_42_ between vehicle-treated (235.8 ± 16.8, *n* = 9) and CBDA-treated (219.4 ± 10.3, *n* = 10) APP/PS1 mice. (**B**) Levels of Aβ_42_ in the aggregated extract were significantly lower in CBDA-treated APP/PS1 mice (13.7 ± 0.7, *n* = 10) than in vehicle-treated control APP/PS1 mice (34.2 ± 2.4, *n* = 9) (*** *p* < 0.001). Data presented as mean ± SEM and analysed using unpaired Student’s t-test.

As we observed an enhancement in LTP in APP/PS1 mice treated with CBDA, we investigated the levels of Aβ in brain tissue from these mice. We used ELISA to assay levels of cortical Aβ_40_ in vehicle-treated and APP/PS1 mice that had received CBDA. We found that there was no significant change in the levels of soluble Aβ_40_ between vehicle- and CBDA-treated APP/PS1 mice across all groups (see Supplementary Materials). We also used ELISA to assay levels of soluble and aggregated Aβ_42_ in cortical tissue between vehicle- and CBDA-treated (30 mg/kg) mice. We found there was no significant difference in soluble Aβ_42_ between the vehicle-treated (235.8 + 16.8 *n* = 9) and CBDA-treated groups (219.4 + 10.4, *n* = 10 (Figure 4A). When the levels of aggregated Aβ_42_ were examined, there was a significant difference between the vehicle-treated APP/PS1 (34.2 + 2.4, *n* = 9) and CBDA-treated APP/PS1 (13.7 + 0.7, *n* = 10) (*p* < 0.001) groups. Values are expressed as pg Aβ/mg protein (Figure 4B).

### 2.5. Mass Spectrometry and Proteomic Analysis

**Figure 5:**
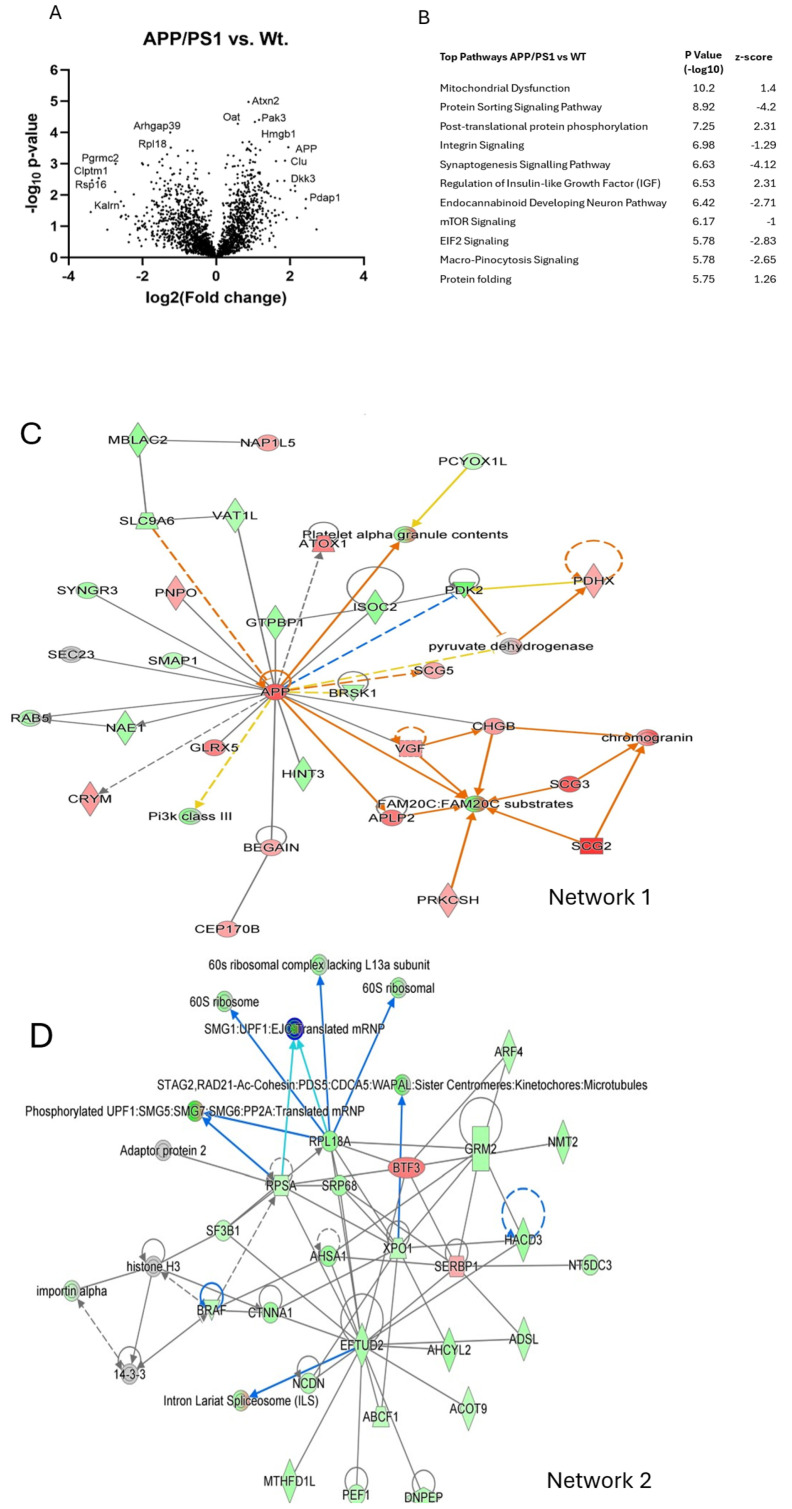

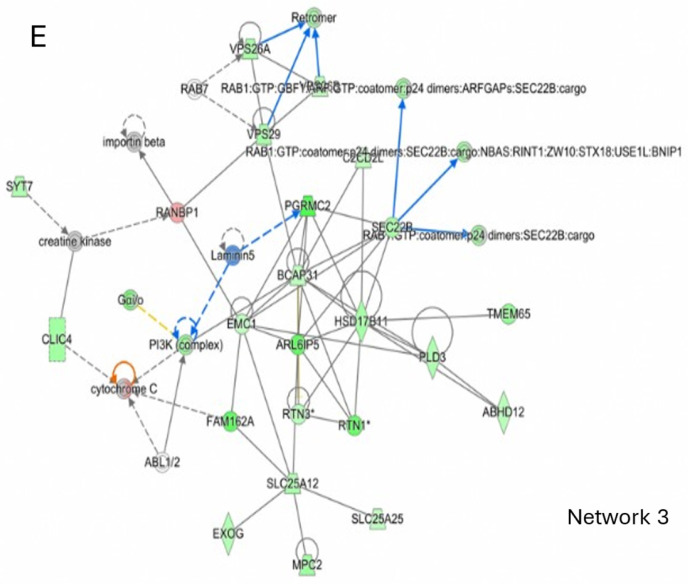
Network and pathway analysis of vehicle-treated APP/PS1 genotype on cortical protein expression levels compared to vehicle-treated WT controls. (**A**) Volcano plot, data presented as Log2 (fold change) versus -Log10 (*p*-value). Statistical significance set using unpaired Student’s t-tests. (**B**) Canonical pathways (ranked by *p*-value; with positive and negative Z-scores predicting activation and inhibition, respectively) highlight predicted inhibition of protein sorting, endocannabinoid, micropinocytosis, and synaptogenesis signalling with increased mitochondrial dysfunction. (**C**) Networks: data were imputed into Ingenuity Pathway Analysis (by Qiagen) and ranked by *p*-value and Z-score. The main enriched network (ranked by *p*-value) altered in APP/PS1 vs. WT mice shows a central node to be APP (which is involved in the predicted activation (orange arrows) of Fam20C proteins and platelet alpha granule release). Of note, increased expression of APLP2 and Scg2 converges to result in predicted activation of Fam-20 substrates. (**D**) The second highest network (ranked by *p*-value) demonstrates decreased expression of Eftud2 (a key spliceosomal protein) regulating other proteins associated with ribosomal function and protein synthesis. (**E**) The third highest network (ranked by *p*-value) highlights endosome to Golgi apparatus protein trafficking (retromer inhibition) in addition to predicted Sec22b inhibition of cargo transport in vesicles which complex with SNARE. See Figure 7 for the prediction legend.

**Figure 6:**
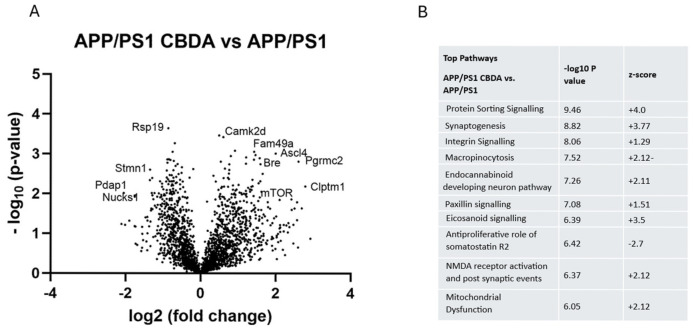

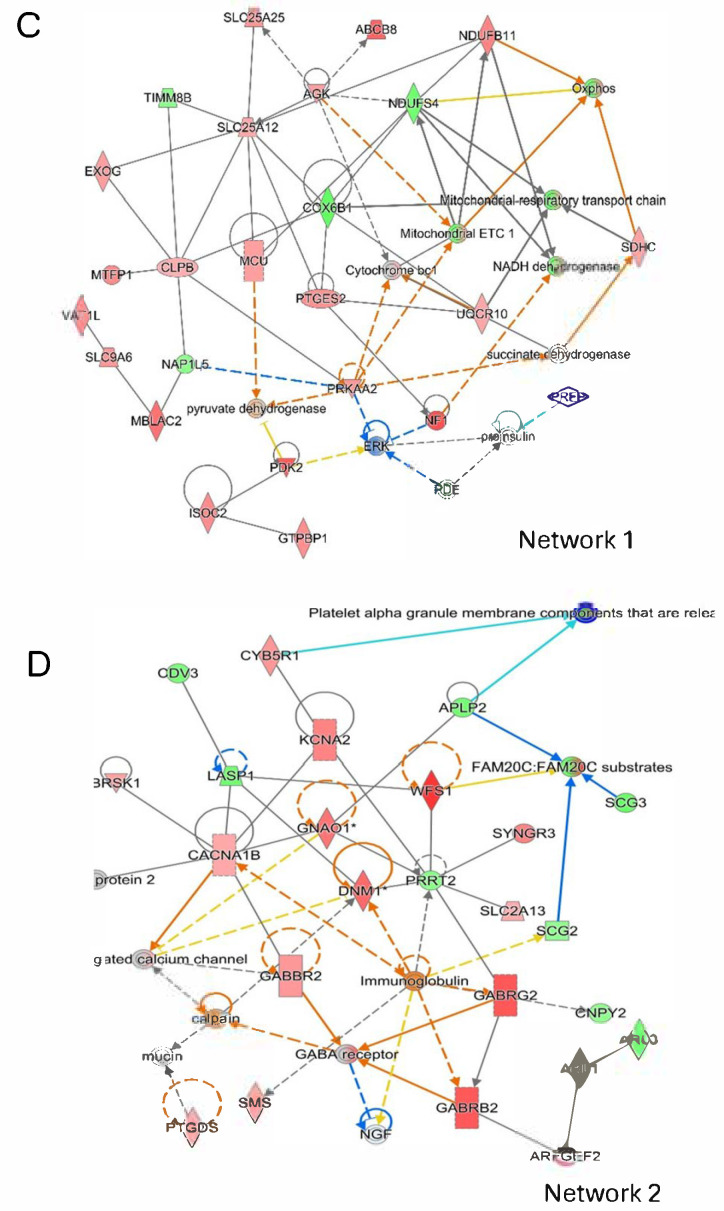
Network and pathway analysis of cortical protein expression in CBDA-treated APP/PS1 compared to vehicle-treated APP/PS1 mice. (**A**) Volcano plot: data presented as log2 (fold change) versus -Log_10_ (*p*-value). Statistical significance set using unpaired Student’s *t*-tests. (**B**) Canonical pathways (ranked by *p*-value, with positive and negative Z-scores predicting activation and inhibition, respectively) show predicted activation of protein sorting, endocannabinoid, macropinocytosis, and synaptogenesis signalling with reduced mitochondrial dysfunction. (**C**) Network 1: data were imputed into Ingenuity Pathway Analysis (Qiagen) and are the output of the pathway analysis. The main enriched network (ranked by *p*-value) altered in APP/PS1 CBDA treated vs. vehicle-treated APP/PS1 mice shows decreased expression of mitochondrial proteins but predicted activation of mitochondrial oxidative phosphorylation and predicted inhibition of both ERK and proinsulin signalling. (**D**) Network 2: within the second highest ranked network, there is predicted inhibition (blue arrows) of both Fam20c proteins and platelet alpha granule release. The second highest network (ranked by *p*-value) demonstrates decreased expression of APLP2, Scg2, and Scg3, resulting in converging predicted inhibition of Fam-20. See Figure 7 for the prediction legend.

**Figure 7:**
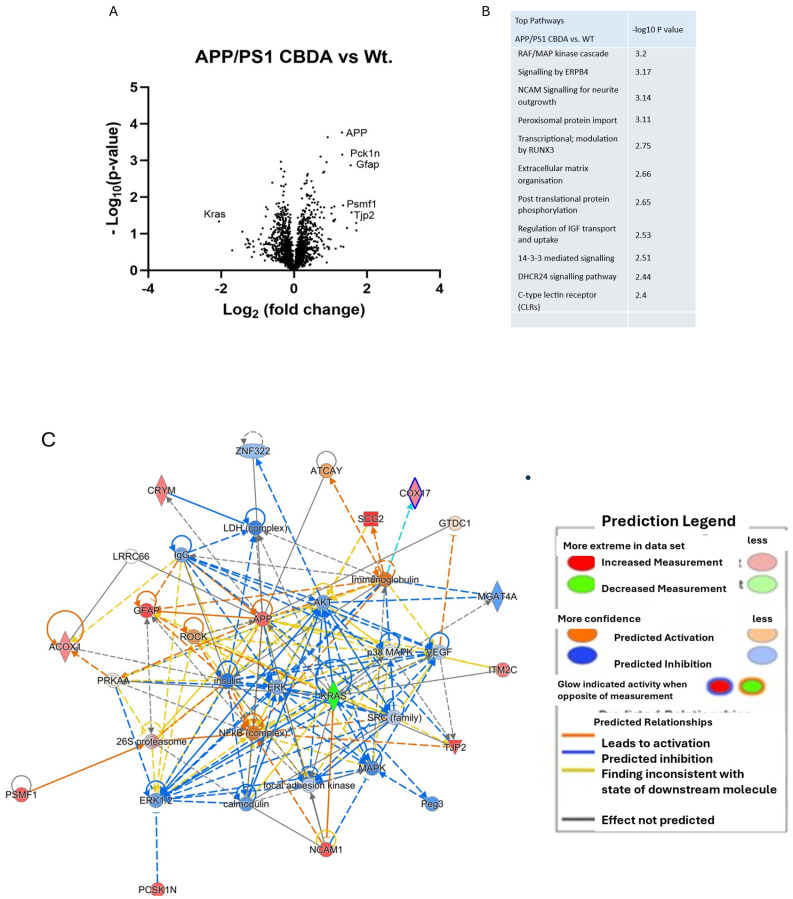
Network and pathway analysis of cortical protein expression in CBDA-treated APP/PS1 mice compared to vehicle-treated WT mice. (**A**) Volcano plot: Data presented as log2 (fold change) versus –Log10 (*p*-value). Statistical significance set using unpaired Student’s t-tests. (**B**) Canonical pathways (ranked by *p*-value) suggest a role for MAP kinase, Epb4, and Ncam signalling in addition to protein import and phosphorylation processing. (**C**) Data were imputed into Ingenuity Pathway Analysis (Qiagen) and are ranked by *p*-value and Z-score. The main enriched network (ranked by *p*-value) altered in CBDA-treated APP/PS1 vs. WT mice shows predicted inhibition of insulin, Akt, MAP kinase, and ERK proteins, while both NfKb and APP are predicted to remain activated. (**C**) Canonical pathways (ranked by *p*-value) suggest a role for MAP kinase, Epb4, and Ncam signalling in addition to protein import and phosphorylation processing.

Mass spectrometry was conducted on the cortex from male WT vehicle-treated (*n* = 4), APP/PS1 vehicle-treated (*n* = 3), and APP/PS1 CBDA (*n* = 4)-treated mice. Significantly changed (*p* < 0.05) proteins (WT+vehicle vs. APP/PS1+vehicle = 370; APP/PS1+vehicle vs. APP/PS1+CBDA = 196; APP/PS1+CBDA vs. WT = 4) were identified using Perseus. Ingenuity Pathway Analysis (IPA) was used to identify enriched pathways, upstream regulators, and associated networks. A list of the top signalling pathways identified as being significantly altered between groups is available in Supplementary Materials as well as the shorter list available in Figure 5 showing the comparison of APP/PS1 (vehicle)-treated vs. WT (vehicle)-treated mice. The comparison between CBDA-treated APP/PS1 mice and APP/PS1 (vehicle)-treated mice is shown in Figure 6. When comparing APP/PS1 CBDA-treated vs. WT vehicle-treated mice (Figure 7), there was a low number of significant proteins emerging, with no Z-scores associated with the potential canonical pathways. Comparative tables are provided of identified proteins and pathways demonstrating the main differences between WT (vehicle-treated) and APP/PS1 (vehicle-treated) (Tables S1 and S2), APP/PS1 (CBDA-treated) vs. APP/PS1 (vehicle-treated) (Tables S3 and S4), and APP/PS1 (CBDA-treated) vs. WT (vehicle-treated) (Tables S5 and S6). Supplementary Materials.

#### APP/PS1 Compared to Wild-Type Cortex

Differentially expressed proteins between the APP/PS1 and WT cortex are shown in the volcano plot (Figure 5A). Significantly upregulated proteins in APP/PS1 included amyloid precursor protein (APP), ataxin2, serine threonine protein kinase (Pak3), high-mobility group protein B1 (Hmgb1), ornithine amino transferase (Oat), tubulin polymerisation-promoting protein (Tppp3), heterogeneous nuclear ribonucleoprotein H2 (HnrnPh2), sorting nexin 3 (Snx3), and 60 K heat shock protein (Hspd1). Downregulated proteins included RhoGTPase-activating protein 39 (Arhgap39), 60S ribosomal protein l18 (Rpl18), 40S ribosomal protein16 (Rpl16), long-chain fatty acid co-A-ligase4 (AcsL4), and kalirin or Huntingtin-associated protein-interacting protein, associated with nerve growth and axonal development (Kalrn). For the top 100 proteins, see Table S1 in the Supplementary Materials.

The three top networks are also shown (Figure 5C–E). These demonstrate the core effects of amyloid precursor protein (APP), central to pathways involved in synapse formation, neural plasticity [32], antimicrobial activity [33], iron export [34], and ribosomal protein production. Figure 5C highlights the central node of APP, which is involved in the predicted activation (orange arrows) of Fam20c proteins and platelet alpha granule release. Furthermore, increased expression of APLP2 and Scg2 converges to result in predicted activation of Fam-20 substrates. Figure 5D also shows decreased expression of Eftud2 (a key spliceosomal protein) regulating other proteins associated with ribosomal function and protein synthesis in APP/PS1 mice. A central role for altered protein trafficking in APP/PS1 mice is also clear, with Figure 5E showing predicted inhibition of the retromer (a component of endosome-to-Golgi apparatus protein trafficking) in addition to predicted Sec22b inhibition of cargo transport in vesicles that complex with Snap receptors (SNAREs).

The top significant signalling pathways are shown in Figure 5B (additional information on pathways is in Supplementary Materials Table S2). The altered canonical pathways in APP/PS1 mice highlight predicted mitochondrial dysfunction in addition to inhibition of protein sorting, endocannabinoid signalling, micropinocytosis, and synaptogenesis signalling. One of the most significant pathways identified is mitochondrial dysfunction in APP/PS1 mice compared to WT, with a high probability and a relatively low z-score. Concerning mitochondrial dysfunction in APP/PS1 cortical tissue, the list of differentially expressed proteins named within IPA analysis as part of this pathway included amyloid precursor protein (APP), cytochrome c oxidase subunit 5A (Cox 5a), cytochrome c oxidase subunit 6A1 (Cox 6a1, complex IV), cytochrome c mobile electron transport carrier (Cycs), complex 1 NADH oxidoreductase subunit 4 (Ndufs4), and pyruvate dehydrogenase (Pdhx). Other differentially expressed proteins that were decreased included phosphatidylinositol 3-kinase catalytic subunit type 3 (Pik3c3), the alpha one subunit of AMP-activated protein kinase (AMPK) (Prkaa1/Prkaa2), succinate dehydrogenase cytochrome b560 (Sdhc), mammalian target of rapamycin (mTOR) and NADH ubiquinone oxidoreductase (complex I) (Ndufb6), glutathione S transferase theta 1 (Gstt1), and dynamin-1-like protein (DnmlL). Some of these changes are summarised in the heat map (Figure S1 Supplementary Materials).

The second most significant pathway was protein sorting and signalling (Figure 5B). A number of significantly differentially expressed proteins were decreased in APP/PS1 mice compared to wild-type controls and include adaptor-related protein complex 1 (Ap1g1), Copi coat complex subunit (Copb1), Arfgef2, and transmembrane p24 trafficking protein 2 and 9 (Tmed2 and Tmed9) (see Figure S2, Supplementary Materials).

In relation to the synaptogenesis signalling pathway in APP/PS1 vs. WT mice, significantly differentially expressed proteins that were decreased included glutamate ionotropic receptor AMPA-type subunit 1 (Gria1), glutamate metabotropic receptor 2 (Grm2), glutamate receptor metabotropic receptor 5 (Grm5), mechanistic target of rapamycin kinase (mTOR), kalirin rho kinase (Kalrn), phosphatidylinositol -3 kinase subunit 3 (Pik3c), Ras-related protein 2 (Ras2), and synaptotagmin (Syt7) (Figure S3). In addition, comparison of differentially expressed proteins involved in integrin signalling are represented in Supplementary Materials (Figure S4).

### 2.6. CBDA Versus Vehicle Treatment in APP/PS1 Cortex

When comparing APP/PS1 CBDA vs. APP/PS1 (vehicle), there was an altered profile of differentially regulated proteins, as shown in the volcano plot within Figure 6A. The high-mobility group protein B1 (Hmgb1) was significantly decreased in expression. Small 40S ribosomal protein (RPs19), eukaryotic translation initiation factor (Eif3i) and small glutamine-rich tetratricopeptiderepeat protein (Sgtb), and ataxin2 were also reduced in expression. In contrast, dipeptidyl amino peptidase-like protein 6 (Dpp6) was increased, as was calcium calmodulin-dependent protein kinase 2 (Camk2d), Lanc-like protein 2 (LancL2), protein family 49a (Fam49a), long-chain fatty acid co-A-ligase4 (AcsL4), and glutamate ionotropic receptor AMPA-type subunit 1 (GRIA1). Of importance is the fact that APP was not significantly differentially expressed between the APP/PS1 CBDA vs. APP/PS1 groups, indicating that CBDA does not act to alter APP expression but instead acts on other biochemical mechanisms.

The top-ranked enriched network (Figure 6C; ranked by *p*-value) altered in CBDA-treated APP/PS1 vs. vehicle-treated APP/PS1 mice showed decreased expression of mitochondrial proteins but predicted activation of mitochondrial oxidative phosphorylation and predicted inhibition of both ERK and proinsulin signalling. Within the second highest ranked network (Figure 6D), there was predicted inhibition (blue arrows) of both Fam20c proteins and platelet alpha granule release which were both activated in the APP/PS1 vs. wild-type mouse comparison. Furthermore, APP/PS1 mice treated with CBDA showed decreased expression of amyloid-like protein 2 (APLP2) and secretogranin proteins 2 and 3 (Scg2 and Scg3), which appear to converge on predicted inhibition of Fam20 kinase (Fam-20) (Figure 6C).

Metabolic and protein signalling pathways were identified as being altered in APP/PS1 CBDA-treated vs. APP/PS1 vehicle-treated mice. A number of significant pathways were reversed in functionality in the APP/PS1 cortex following CBDA treatment compared to vehicle-treated APP/PS1 mice. Mitochondrial dysfunction, synaptogenesis, and protein folding pathways were reversed in the direction of effect, with mitochondrial dysfunction being inhibited, while synaptogenesis and protein folding signalling both demonstrated activation following CBDA treatment in APP/PS1 mice (Figure 6B).

The most significant pathway was protein sorting and signalling, which had a change in expression directionality, showing increased levels of all the proteins listed previously, with Arfgef2, Ap1g1, and Tmed2 significantly differentially expressed. The second highest (ranked by *p*-value) pathway was synaptogenesis, in which proteins that were decreased in APP/PS1 compared to wild-type mice were all increased in expression in the APP/PS1 mouse cortex following CBDA treatment (Figure S3). When proteins associated with mitochondrial dysfunction pathway were examined following CBDA treatment, we found a decrease in Ndufs4, cytochrome c oxidase (Cox5A), and Sdha (succinate dehydrogenase flavo-protein subunit), while mammalian target of rapamycin (mTOR) was significantly increased.

### 2.7. CBDA-Treated APP/PS1 Mice Versus Vehicle-Treated Wild-Type Control Mice

Following CBDA treatment in APP/PS1 mice compared with vehicle-treated wild-type mice, there was a decrease in expression of proteins coded by Hmgb1, Dkk3, Rsp19, and Pdap1, while amongst other proteins, including Clptm1, Pgrmc1, FAAH, Camk2d, and Kalrn expression was increased. The number of differentially expressed proteins was lower than that of other group comparisons discussed earlier. A comparative table of the top proteins altered in APP/PS1 (vehicle) vs. WT (vehicle) and APP/PS1 CBDA vs. APP/PS1 vehicle is available in the Supplementary Materials showing protein expression changes denoted both by *p*-value and log_2_ difference.

Of note, CBDA-treated APP/PS1 vs. WT mice showed predicted inhibition of insulin, AKT, MAP kinase, and ERK proteins, while APP was predicted to remain activated (Figure 7C). This reinforces the concept that amelioration of the effects of APP in APP/PS1 mice by CBDA is not due to significantly altered APP expression.

Analysis of the canonical pathways for significant differential cortical protein expression in CBDA-treated APP/PS1 mice compared to vehicle-treated wild-type controls is shown in Figure 7B. The canonical pathways (ranked by *p*-value) suggest a role for MAP kinase, Erpb4, and Ncam signalling in addition to protein import and phosphorylation processing, but no direction of effect (activation or inhibition) could be predicted, meaning no Z-scores are reported due to the relatively small number of differentially expressed proteins input into the pathway analysis. Additional information is available in Supplementary Materials Table S3.

While comparing wild-type, APP/PS1, and CBDA-treated APP/PS1 cortex using Ingenuity analysis, four key pathways emerged in which individual protein expression changes were evident, as shown in the heat maps presented in Supplementary Materials Figures S1–S4. These reflect mitochondrial proteins (Figure S1) and proteins involved in protein sorting (Figure S2), synaptogenesis (Figure S3), and integrin signalling (Figure S4).

## 3. Discussion

CBD and THC have been the focus of many studies by the scientific community and clinicians alike [35]. The predominant cannabinoids in hemp, however, are THCA and CBDA [36]. CBDA is known to cross the blood–brain barrier and to penetrate the brain [37]. In support of its therapeutic benefits, CBDA application in animal models has been shown to have anti-hyperalgesia [19,20], anti-seizure [21], anti-inflammatory [22], and anti-emetic properties [38]. CBDA has also been shown to rescue object and spatial cognitive function and memory deficits in Aβ_42_-treated mice [26]. CBDA is, however, the chemical precursor to CBD [21,28,29], which has also been shown to have many therapeutic properties. The possible effects mediated by this metabolite therefore cannot be dismissed. Long-term treatment with CBD has also been shown to prevent the development of social recognition memory deficits and improve object recognition in APP/PS1 mice [39,40]. CBDA is known to have increased oral absorption compared to CBD, with a serum concentration 30 times higher than that of CBD following oral doses of 2 and 4 m/kg [41].

Here, we have shown for the first time that the deficit in LTP produced by acute application of Aβ_42_ (amyloid-derived diffusible ligands) [11,42,43] can be reduced by prior treatment with acute application of CBDA. We have also shown that acute CBDA did not significantly alter the level of LTP or PTP, similar to our previous observations with CBD [11]. We used only one concentration of CBDA (10 μM) and therefore cannot speculate as to the potential dose dependency of the effect. In a previous study using the same model, we found that the protective effects of CBD at the same concentration were reversed by the PPARγ antagonist GW9662 [11]. Here, we also demonstrated a similar reduction in the acute protective effects of CBDA against acute Aβ when PPARγ was inhibited. As CBDA has also been shown to activate PPARγ [44], the acute protective effects mediated here are likely mediated via this signalling cascade. The PPARγ nuclear receptor in the CNS is expressed in low levels, increasing under inflammatory conditions. PPARγ activation causes inhibition of NFkB to decrease expression of inflammatory genes [45]. In addition, CBDA has been shown to inhibit the expression and activity of cyclooxygenase 2 (COX-2) [25,46], further enhancing its anti-inflammatory effects. Most importantly, CBDA has been shown to be a powerful antioxidant comparable to vitamin E [24].

While we demonstrated the effects of CBDA in vitro against Aβ_42_, the next step was to examine possible effects of CBDA in both control WT mice and in a transgenic model of AD (APPswe/PS1dE9; APP/PS1). There were no data available on the chronic effects of CBDA on synaptic transmission in either WT or APP/PS1 mice. In agreement with our previous report, LTP in the CA1 region in slices from APP/PS1 mice was depressed compared to levels in slices from vehicle-treated non-transgenic littermates [13]. While there was a slight trend towards increased LTP levels in slices from non-transgenic WT littermates with increasing doses of CBDA, these changes were not significant compared to those recorded in slices from vehicle-treated mice. This suggests that CBDA does not alter synaptic LTP and is not detrimental to plasticity in WT mice. Chronic treatment of APP/PS1 mice with CBDA at all concentrations, however, reversed the deficit we observed in LTP recorded in vehicle-treated APP/PS1 mice. This could be due to the alterations we observed in proteins involved in synaptogenesis and synaptic function (see the proteomics discussion).

While our focus was on the effects of CBDA on LTP, the possible overall change in excitability was also of interest. CBD and CBDA have been proposed to act as antiseizure agents [21] and could possibly have altered neuronal excitability. Short-term changes in excitability were monitored using PPF at a range of inter-pulse intervals (10–150 ms). We observed a deficit in PPF in slices from vehicle-treated APP/PS1 mice in contrast to other studies that have shown little change in paired-pulse ratio in the hippocampal CA1 region of APP/PS1 mice [47]. We found that CBDA could reverse this deficit. It has been proposed that in aging synapses there is an increase in resting presynaptic calcium following Schaffer collateral stimulation, accompanied by a decrease in PPF [48]. Cannabinoids have been reported to inhibit T-type calcium channels, with CBDA having a higher efficacy than CBD, which could influence neurotransmitter release [49]. The reduction in PPF in slices from APP/PS1 mice may suggest higher levels of residual presynaptic calcium related to increased tonic release. Alternatively, the previously reported decrease in L-type channel density in APP/PS1 mice may be involved [50]. The effects we observed are highly unlikely to be due to residual CBDA present in slices, as the experiments were conducted 48 to 72 h following the last IP injection. The half-life of CBDA has been reported to be less than 4 h in mice [37].

In a study of hippocampal autapses, CBDA was found to have little effect on neuronal cannabinoid receptors. CBDA did, however, increase calcium in 2% of dorsal root ganglia suggesting it may act via a TRPV1 activation [51]. If CBDA was metabolised to CBD in vivo, then it could influence depolarisation-induced suppression of excitation [52], consistent with CBD acting as a negative allosteric modulator (NAM) at CB1 receptors [53]. In a study on the binding properties of CBDA, it was shown to improve the formation of heteromers of CB1R and CB2R, while CBD decreased affinity. In addition, CBDA affinity was in the micromolar range in radioligand-based assays using either CB1R or CB2R-containing membranes, higher than CBD. It was also suggested that CBDA may act as an inverse agonist or NAM at both CB1R and CB2R [54]. If CBDA was decarboxylated to CBD [55], the latter could reduce the uptake of anandamide to influence the ECB system via inhibition of fatty acid amide hydrolase, but this would require a high concentration of CBD since the IC_50_ in rat tissue is 15.2 μM [56]. While inhibition of anandamide breakdown has been shown to restore LTP in a model of osteoarthritis via activation of CB1R [57], others have shown that elevation of endogenous anandamide impaired LTP [58]. The alterations in PPF and LTP we observed were likely due to the major alterations in proteins associated with mitochondrial function and synaptogenesis, many of which were rescued by CBDA treatment (see the proteomics discussion). We investigated the possibility that CBDA treatment could reduce the level of either soluble or aggregated Aβ. The levels of soluble Aβ_40_ were similar in CBDA-treated and vehicle-treated APP/PS1 mice (see Supplementary Materials). Reported levels of Aβ_40_ and Aβ_42_ vary across the literature; a previous report shows lower levels of soluble Aβ_40_ and Aβ_42_ at 8–9 months in APPswe/PS1dE9 brain extract [59], while another study reported a much higher value for aggregated Aβ_42_ at 5 and 12 months [60]. Our data demonstrating a decrease in the level of aggregated Aβ_42_ were significant in mice treated with CBDA (30 mg/kg); however, the level of Aβ_42_ that we measured was lower than predicted. Our data demonstrating a decrease in the level of aggregated Aβ_42_ suggest that CBDA may be acting in a similar manner to THC. The psychoactive cannabinoid agonist THC has been shown to directly interact with Aβ peptide, inhibiting aggregation [61]. Alternatively, assuming decarboxylation of CBDA to CBD, the latter has been shown to enhance microglial phagocytosis of beta amyloid via TRPV2 [62]. This may influence levels of aggregated peptide.

Mass spectroscopy/proteomic analysis on cortical tissue was conducted to determine potential metabolic and signalling pathways that were significantly different between vehicle-treated wild-type and APP/PS1 mice. We were also interested to determine whether CBDA treatment (10 mg/kg) altered any of these pathways in the APP/PS1 cortex of APP/PS1 mice.

Comparing the canonical pathways that were significantly different between APP/PS1 mice and WT controls highlighted mitochondrial dysfunction and processes, including protein sorting and signalling, post-translational protein phosphorylation, integrin signalling, and synaptogenesis.

Mitochondrial Dysfunction: Mitochondria are the primary location for ATP production via the electron transport chain, acetyl coenzyme A (acetyl-CoA) oxidation, and oxidative phosphorylation. Cellular processes, including cell adhesion, synaptogenesis, protein sorting, and neurotransmitter release, require energy expenditure [63] depending on the metabolism of ATP and adenosine diphosphate (ADP). Considering mitochondrial dysfunction, of the top 25 proteins suggested to be involved in the IPA pathway analysis, 11 were significantly differentially expressed in the APP/PS1 cortex, with the others acting as connecting elements between those that were differentially expressed. Amongst those that were upregulated were APP, Cox5a, Cox6a1, Cycss, Ndufs4, and PDHX, while those that were downregulated included Sdhc, Prkaa1, Prkaa2, Pik3c3, mTOR, Gstt1, and Dnajc11 in APP/PS1 mice compared to wild-type controls (shown in the heat map in the Supplementary Materials. Considering how these proteins can alter function, these involve changes along the mitochondrial electron transport chain, with increases in Cox5a (cytochrome c oxidase subunit 5A, complex IV) and Ndufs4 (NADH oxidoreductase subunit 4) potentially increasing levels of reactive oxygen species, leading to oxidative stress and cellular damage. In addition, Cycs (a mobile electron transport carrier), NDUFS4, and PDHX (pyruvate dehydrogenase) can all increase ATP production, which can be used both as an energy source and as an agonist at purinergic receptors such as the pro-inflammatory P2X7 receptor. In the case of PDHX, its upregulation leads to increased levels of acetyl-CoA, which is a key substrate of the Krebs cycle to increase ATP production. Downregulation of Sdhc (succinate dehydrogenase) may impair complex II function, leading to reduced electron transport from succinate to ubiquinone and to diminished mitochondrial electron transport chain activity. Decreased production of fumarate from succinate could also impair the TCA cycle and thereby ATP production. There are also changes in the ATP sensor AMPK (AMP-activated protein kinase) with downregulation of Prkaa1/2 protein encoding the alpha one subunit of AMPK, which regulates cellular glucose and lipid metabolism. Upon AMPK activation, it phosphorylates and inactivates acetyl-CoA carboxylase (ACC) and beta-hydroxy beta-methylglutaryl-CoA reductase (Hmgcr), key enzymes involved in regulating de novo biosynthesis of fatty acids and cholesterol. Studies suggest that this catalytic subunit may control whole-body insulin sensitivity and is necessary for maintaining insulin resistance [64]. Interestingly, the altered expression of some of these mitochondrial proteins in APP/PS1 were reversed by CBDA, with the mitochondrial dysfunction pathway also showing less activation following CBDA treatment in the APP/PS1 mice. Mitochondrial dysfunction has been noted previously in APP/PS1 mice [65,66].

Beyond its partial reversal of protein expression involved in mitochondrial dysfunction, treatment with CBDA appears to alter protein expression in complexes I, II, and IV of the electron transport chain, which may increase activity within the electron transport chain in CBDA-treated APP/PS1 mice. For example, Cox5a (cytochrome C oxidase subunit 5A), a component of cytochrome C oxidase, is a member of complex IV, drives oxidative phosphorylation, and may increase ROS production. Its expression was increased in APP/PS1 mice, which was reversed by CBDA. Interestingly, Cox5a, which was rescued by CBDA in our study, has been reported to impact LTP. Transgenic mice with systemic Cox5a overexpression demonstrated improvement in hippocampal synaptic plasticity [67] Conversely, it has been suggested that NMDAR-dependent LTP induction causes a rapid burst of dendritic mitochondria fission, which, when blocked, prevents LTP [68]. This suggests that some of the mitochondrial proteins rescued by CBDA could impact LTP by altering mitochondrial fission through their regulation of DRP1 (dynamin related peptide 1), a cytosolic dynamin GTPase crucial for mitochondrial fission [69].

Protein Sorting: Protein sorting as a process involves both (i) signal-based sorting, where amino acids act as sorting signals, facilitating either gated transport or translocation of protein targets, and (ii) vesicle-based trafficking at the Golgi apparatus (GA), plasma membrane, or endosomes. There are clear changes in protein sorting (on a pathway level but also on an individual protein level) in the cortex of the APP/PS1 mice. Amongst those significantly downregulated were adaptor-related protein complex 1 subunit gamma 1 (Ap1g1), ARF guanine nucleotide exchange factor 2 (Arfgef2), coatomer protein subunit beta (Copb1), and transmembrane p24 trafficking protein 2 and 9 (Tmed2 and Tmed9) in APP/PS1 mice compared to wild-type controls (see the heat map in the Supplementary Materials). Downregulation of Ap1gp1 impacts protein trafficking within the trans-Golgi network and endosomes, while variants of Ap1g1 can cause neurodevelopmental abnormalities [70]. Arfgef2 plays an important role in intracellular vesicular trafficking, with mutations being associated with neural migration disorders [71]. The coatomer protein (Copb1) is required for budding from the GA interacting with GTPases encoded by the GTP-binding protein Sar1A. Both Copb1 and Sar1 are critical to retrograde transport between the endoplasmic reticulum (ER) and the GA. Interestingly, polymorphisms in coatomer associated proteins have been shown to be associated with increased Alzheimer’s disease risk [72]. The transmembrane domains (Tmed) Tmed2, and Tmed9 are glycoproteins that are present on the ER and involved in protein trafficking to the Golgi apparatus. Notably, Tmed2 plays a role in vesicular transport and regulates both interferon and Toll-like receptor signalling. Both Tmed9 and Vps29 have a role in autophagy, while the vacuolar protein sorting (Vps) proteins interacting with the retromer protein complex are involved in retrograde transport from the endosome to the trans-Golgi network (reduced in the heat map). The regulation of interferon and Toll-like receptor signalling via action on protein sorting processes in APP/PS1 may play a role in neuroinflammation.

CBDA treatment caused a significant increase in and reversal of expression of Tmed2, Tmed7, Copb1, Ap1g1, and Arfgef2. Consequently, this reversal should improve communication and protein trafficking to help restore function in the APP/PS1 cortex towards the control levels observed in WT mice. It should also restore Toll-like receptor trafficking to regulate inflammation and neurodegeneration the APP/PS1 cortex [73].

Synaptogenesis: The ability to form new synapses is key to the maintenance of neuronal health. A number of synaptogenesis-associated proteins were altered in expression in the APP/PS1 mouse cortex compared to wild-type controls. Amongst those significantly downregulated included metabotropic glutamate receptor 2 (Grm2), metabotropic glutamate receptor 5 (Grm5), kalirin RhoGEF kinase (Kalrn), serine/threonine-protein kinase mTOR (mTOR), Ras-related protein Rab-5A (Rab5a), neuronal proto-oncogene tyrosine-protein kinase Src (SRC), Ras-related protein R-Ras2 (Rras2), phosphatidylinositol 3-kinase catalytic subunit type 3 (Pik3C3), Cacna1b (calcium voltage-gated channel subunit alpha1 B), and synaptotagmin-7 (Syt7) (see the heat map in the Supplementary Materials. Considering the function of these proteins, the decreased expression of Grm2 and Grm5 will directly affect neurotransmission and synaptogenesis. Kalrn activates rho GTPases, impacting neuronal shape, growth, and plasticity via the actin cytoskeleton, while mTOR (mammalian target of rapamycin) activation is reported to be associated with increased synaptogenesis [74]. Downregulation of mTOR, in contrast, may reinforce the pathway analysis, showing decreased synaptogenesis in APP/PS1 mice, an effect reversed by CBDA. Syt7 regulates vesicular docking at the plasma membrane [75] and is crucial for normal synaptic transmission. The decreased expression of Cacna1B in the APP/PS1 cortex is also likely to impact neurotransmitter release. compromising synaptic transmission and consequently, reducing plasticity.

CBDA treatment of APP/PS1 mice significantly reversed the expression of many of these proteins. Fundamental proteins Grm5, Kalrnm, Src, and PikK3c3 were all significantly upregulated by CBDA treatment, while Rras2, Cacna1b, and Syt7 had increased expression, but these were not significant. This demonstrates that CBDA could significantly improve synaptogenesis in the APP/PS1 cortex, changing protein expression toward the levels in wild-type controls. These changes are likely to be directly linked to improved levels of LTP and PPF in APP/PS1 following CBDA treatment.

Integrin Signalling:Alterations in integrin signalling links directly to alterations in synaptogenesis [76] via decreased synaptic protein expression, with decreases in Gria1 (AMPA receptor subunit 1), Grm2 (glutamate metabotropic receptor2), and Grm5 (glutamate metabotropic receptor 5). There is also a notable decrease in Kalrn (Kalirin), the synaptic regulator [77] that is known to be reduced in expression in the hippocampus of Alzheimer’s disease patients [78]. Integrins are important structural transmembrane receptors involved in adhesion between cells and to the extracellular matrix [79] but also play an important role in neurite growth [80]. With regard to integrin protein expression changes, amongst those that were significantly upregulated were Cttn (Src substrate cortactin) and Pak3 (p21 activated kinase, the target receptor of paxillin, a prototypical integrin), while those that were downregulated included ARF GTPase-activating protein (Git1), Ras-related protein 2A (Rab2A), Ras-related protein R-Ras2 (Rras2), and tetraspanin-7 (Tspan7) in APP/PS1 mice compared to wild-type controls. The decrease in Git1 in APP/PS1 may result in decreased spine density and spine development [81], while the decrease in the endocytotic membrane protein Tspan7 is likely to interrupt the integrin beta1/Fak/Src signalling pathway [79], with Src also being reduced. Decreased Tspan7 is involved in spine maturation and AMPA receptor trafficking [82], and it also interacts with the metalloprotease ADAM10, which can regulate both presenilin and amyloid precursor protein [83]. Decreased Pik3c3 in APP/PS1 cortical tissue is likely linked to decreased plasticity. Pik3c3 is widely expressed in neurons, producing PI3P in dendritic spines. Pik3c3 is also required for mTOR signalling [84]. Deletion of Pik3c3 leads to a loss of synapses in cortical pyramidal neurons, gliosis, and neurodegeneration [85]. The increase in Pak3 is known to be specifically involved in FAD APP-mediated neuronal apoptosis, in which the serine–threonine kinase interacts with APP [86].

While we noted a decrease in v-raf murine sarcoma viral oncogene homolog B1 (Braf) in APP/PS1, there was an increase above levels observed in the WT cortex following CBDA treatment. As Braf is known to be associated with cell division and differentiation linked to ERK/MAP kinase, this may also drive improved synaptic plasticity. Integrin signalling is key to new synapse formation [87], and it is clear from the canonical pathway analysis that this was inhibited in APP/PS1 mice but improved following CBDA treatment. This has important consequences for the capacity to signal not only at synapses but also at dendritic spines and neurites, likely to help rescue synaptic plasticity and influence paired-pulse facilitation. However, the molecular machinery to support integrin signalling was diminished in the APP/PS1 mice, as a number of key proteins were downregulated. While CBDA treatment improved the directionality of protein expression of many proteins associated with integrin signalling in the integrin, the proteins Cttn, Pak3, Src, and Pik3c3 were significantly increased.

All of the above changes in mitochondrial, synaptogenesis, protein sorting, and integrin signalling protein expression were improved by the administration of CBDA to the APP/PS1 mice. Given the consequences of decreased ATP production for energy-dependent processes such as protein sorting and synaptogenesis, it is of no surprise that mitochondrial change may underline the synaptic plasticity changes produced by CBDA in the APP/PS1 mice, but this remains to be experimentally tested.

In addition, the network analysis of the proteome of the APP/PS1 mouse cortex compared to that of the WT controls suggests a central node to be APP and predicted activation of Fam20c proteins and platelet alpha granule release. Increased expression of APLP2 and Scg2 converged to result in predicted activation of Fam-20 substrates. Taken together, this suggests a pro-inflammatory phenotype in the APP/PS1 mouse cortex. Interestingly, treatment with CBDA inhibited both Fam20c proteins and platelet alpha granule release without changing APP tone per se. This suggests that both Fam20 inhibition and the inhibition of platelet alpha granule release play a role in anti-inflammatory actions of CBDA in the APP/PS1 mouse cortex. Decreased expression of Eftud2 (a key spliceosomal protein) was also observed in the APP/PS1 mice, which confirms the pathway analysis of altered protein processing. Edtud2 regulates other proteins associated with ribosomal function and protein synthesis and is reported to be brain protective [88]. Its decreased expression in APP/PS1 mice may underlie in part the reduction in synaptic plasticity and neurodegenerative AD-like phenotype. The network analysis also confirmed the altered protein sorting processing seen in the pathway analysis. In particular, it highlights endosome-to-Golgi apparatus protein trafficking (retromer inhibition) in addition to Sec22b inhibition of cargo transport in vesicles that complex with SNAREs [89]. CBDA treatment appeared to change protein expression, reverting towards wild-type. All of the above changes in mitochondrial, synaptogenesis, protein sorting, and integrin-signalling protein expression were also seen on a pathway level and were improved by the administration of CBDA to the APP/PS1 mice.

Of the additional proteins upregulated in APP/PS1 mice, high-mobility group box protein one (Hmgb1) is a nuclear protein known to be released by glial cells in AD to initiate neuroinflammation. Hmgb1 functions as an archetypal alarmin and a typical damage-associated molecular pattern (DAMP) molecule signalling via RAGE and TLR4 to increase inflammation and alter cognition [90]. Treatment with CBDA reversed the increase in HMGB1, suggesting a powerful anti-inflammatory action. We also found an increase in the RNA metabolism gene Hnrnph2, in agreement with other studies of AD [91]. Also increased in APP/PS1 was HSpd1 or HSp60, a member of the heat shock protein family essential for the folding and assembly of newly imported proteins in the mitochondria. This has been shown to be upregulated in sporadic and familial AD cortices [92]. This was significantly reversed by CBDA treatment. Also of interest is dimethylargininase (Ddah2), a zinc protein involved in nitric oxide synthase regulation, which is elevated in neurons displaying cytoskeletal abnormalities and oxidative stress in Alzheimer’s disease (AD) [93]; it was also increased in APP/PS1 and decreased significantly following CBDA. Considering the endocannabinoid system, there was downregulation of FAAH (fatty acid amide hydrolase), which is known to be involved in the degradation of anandamide in APP/PS1 cortical tissue. FAAH inhibitors have been proposed as potential agents for AD therapy, as genetic inactivation of FAAH in an AD mouse model restored decreases in PPF and LTP [94]. While CBDA restored LTP in our model, it caused an increase in FAAH.

In a previous investigation of the proteomic and lipidomic profile of APPswe/PS1dE9 mice, [95] suggested alterations in both the metabolic and glycerophospholipid profiles at the age of 7 to 8 months. Oxidative stress has been shown to be involved in early memory deficits in APPswe/PS1dE9 mice, linked to decreased levels of glutathione peroxidase and superoxide dismutase [96]. Mitochondrial dynamics are known to be altered in APPswe/PS1dE9 mice, with increased levels of fission and fusion proteins commencing at 3 months, which precede the onset of memory decline [97].The CBDA-mediated improvement in mitochondrial function and synaptogenesis is likely to be key to our observations of improved synaptic plasticity in the form of LTP and short-term changes in transmitter release measured via PPF. Caution should be exercised with regard to the proteomics data interpretation, as (i) the sample size is modest and (ii) this will require independent proteomics confirmation by Western blot of the indicated protein changes and their downstream signalling cascades. It does, however, point towards key mechanisms for further validation.

In summary, we demonstrated that CBDA treatment acts to improve synaptic plasticity and regulate the expression of some essential proteins in the APP/PS1 cortex. CBDA, which has good bioavailability, may provide an alternative approach for the treatment of Alzheimer’s disease and warrants further research.

## 4. Materials and Methods

### 4.1. Mice

Mice were housed 2–5 per cage in a temperature-controlled specific pathogen-free animal facility maintained on a 12 h light/dark cycle (on 7 a.m./off 7 p.m.). The mice were provided with food and drinking water ad libitum. All animal procedures and experiments were approved by the university Animal Research Ethics Committee and performed under a licence from the Health Products Regulatory Authority of Ireland. Initial experiments were conducted using 8–10-week-old male and female C57BL6 mice obtained from Charles River UK to examine the acute effects of CBDA. In addition, we prepared hippocampal slices from 9-month-old APPswe/PS1dE9 (APP/PS1) heterozygote mice bred in our colony within the Biomedical Facility at UCD. Female wild-type C57BL6 mice were crossed with male APP/PS1 mice on a C57BL6 background that was obtained from Jackson Laboratories. These mice have two transgenes inserted at a single locus. The APP Swedish mutation increases the total amount of Aβ produced, and the PS1 sequence lacks Exon 9 (dE9), which increases the relative amount of Aβ_42_ compared to Aβ_40_ [98]. For the ex vivo studies, 9-month-old male and female APP/PS1 mice and their WT littermates were used for hippocampal slice preparations and subsequent biochemical analysis.

### 4.2. Genotyping Procedures

DNA was extracted from ear tissue samples, and the presence of transgenes was confirmed by PCR. For further details, see Supplementary Materials.

### 4.3. Hippocampal Slice Electrophysiology

Parasagittal hippocampal slices (400 μm thick) were prepared using a vibratome (Leica VT1000S), as described previously [11]. Cutting solution was chilled on ice and comprised (mM) NaCl 87, NaHCO_3_ 25, glucose 25, sucrose 75, KCl 2.5, NaH_2_PO_4_ 1.25, CaCl_2_ 0.5, and MgSO_4_ 7, bubbled with 95% O_2_/5% CO_2_ (carbogen). The slices were immediately transferred to a holding chamber containing recording artificial cerebrospinal fluid (aCSF) composed of (mM) NaCl 119, NaHCO_3_ 26.2, glucose 11, KCl 2.5, NaH_2_PO_4_ 1, CaCl_2_ 2.5, and MgSO_4_ 1, bubbled with carbogen, and were allowed to recover for at least 90 min at room temperature. The slices were then transferred to a recording chamber, secured by means of a harp with fine nylon strings, and perfused with recording aCSF at a rate of 4–5 mL/min and maintained at 28–30 °C for the duration of all experiments. Recording electrodes (2–5 MΩ) were filled with recording aCSF and pulled from borosilicate capillary glass (GC150 F-10, Harvard Apparatus) using a horizontal puller (DMZ universal puller; Werner Zeitz, Zeitz-Instruments Vertriebs GmbH**82152**, Martinsried, Germany). The Shaffer collateral pathway was stimulated using a mono-polar electrode (FHC Inc, Bowdoin, ME, USA) at 0.033 Hz (duration: 100 μs); the return electrode was a silver/silver chloride wire placed in the recording bath. Extracellular field excitatory post-synaptic potentials (fEPSPs) were recorded in the CA_1_ *stratum radiatum*, and paired stimuli were delivered with an inter-stimulus interval of either 50 ms or a range of intervals (10–150 ms) to monitor paired-pulse facilitation (PPF). The recorded voltage signal was filtered at 5 kHz and digitised using an Axon Instruments Digidata 1440 A/D board and pClamp10 (Molecular Devices). Signals were amplified by an HS2A Head Stage (Molecular Devices, 3860 N First Street, San Jose, CA, USA) connected to an Axoclamp2B system (Molecular Devices), and a Brownlee 410 Precision preamplifier or an Axopatch1D amplifier. Throughout all experiments, the stimulus voltage was adjusted to evoke a fEPSP that was 40–50% of the maximal response (maximum fEPSP just prior to the formation of a spike caused by cell firing). When a stable baseline had been recorded for 20 min, a Master 8 (AMPI) timer was used to deliver two trains of high-frequency stimuli (HFS) at 100 Hz for 1 s, with an inter-train interval of 30 s. Following the application of HFS, fEPSPs were recorded for a further 60 min. All results are presented as mean ± SEM. The numbers quoted refer to the number of slices used. Control and test experiments in any given section were conducted on the same day on slices from the same animal. Experiments on transgenic mice refer to numbers of mice. Post-tetanic potentiation (PTP) was measured over a 2 min period immediately following the application of HFS.

Amyloid-derived diffusible ligands (ADDLs) Aβ_42_ were prepared as described in Supplementary Materials.

CBDA was provided by Jazz Pharmaceuticals Research UK Ltd. Oxford Business Park, Oxford, OX4 2RW (formerly GW Research UK Ltd., Histon, UK), stored as a stock solution in DMSO (−20 °C) and applied at a concentration of 10 μM for 30 min prior to induction of LTP or application of Aβ_42_. Prior to LTP induction, Aβ_42_ was applied for 30 min at 500 nM. GW9662 was obtained from Tocris Bioscience, and stored as stock in DMSO and at −20°C.

### 4.4. Chronic Treatment with CBDA

The mice were assigned to 8 treatment groups comprising APP/PS1 or wild-type (WT) littermates receiving either vehicle or CBDA (1, 10, or 30 mg/kg). Three doses of CBDA were therefore administered across specific groups of animals at any one time. Male and female APP/PS1 mice and their WT littermates were treated at 8 months by daily intraperitoneal (i.p.) injections for 5 weeks. CBDA was diluted (maximum dilution concentration of 10 mg/mL) in vehicle: ethanol, kolliphor EL, and saline (ratio: 2:1:17). Mouse weights were recorded daily, and animals were euthanised 48–72 h after the final injection. Experiments were conducted over a period of several months.

### 4.5. Protein Extraction for Enzyme-Linked Immunosorbent Assay (ELISA)

Brain tissue was stored at −80 °C, thawed on ice, dissected, and weighed. For Aβ extraction, tissue was homogenised by a 20 s sonication in ice-cold PBS containing protease inhibitor, and BCA was used to determine total protein concentration. Samples were centrifuged at 60,000 rpm for 30 min at 4 °C using an MLA-150 rotor in an Optima Max Ultracentrifuge (Beckman Coulter, Brea, CA, USA). The supernatant (soluble extract) containing soluble, non-plaque-associated Aβ was removed and stored at −80 °C. The pellet was then re-suspended in guanidine hydrochloride 5M (in PBS) and mixed by rotation at room temperature overnight. Finally, following a 20 s sonication, the resulting mix, which contained plaque-associated Aβ, was also stored at −80 °C. Extracts of both the soluble and the extracted aggregated Aβ were used to measure levels of human Aβ in brain tissue by enzyme-linked immunosorbent assay (ELISA).

### 4.6. Aβ ELISA

Brain tissue concentrations of Aβ_40_ and Aβ_42_ were determined using commercially available human amyloid beta 40 and human amyloid beta 42 ELISA kits (ELISA Genie, Dublin, Ireland), performed as per the manufacturer’s guidelines. Diluted samples and standards were loaded in duplicate onto a 96-well plate and incubated for 90 min at 37 °C. The absorbance was measured immediately using the Spectramax M3^®^ (Molecular Devices) plate reader at 450 nm, and Aβ concentrations were determined using the standard curve. The results were finally normalised to the protein concentration, as previously determined by BCA.

### 4.7. Mass Spectrometry Tissue Preparation

All chemicals and solvents used were of proteomic or LC-MS grade unless otherwise stated.

### 4.8. Sample Preparation

Tissue was homogenised in urea lysis buffer (4 cortical samples per group) (6.6 M-urea in 0.05 M triethylammonium bicarbonate buffer (TEAB)), pH 8.5, containing protease inhibitors (cOmplete^TM^ Mini EDTA-free Protease Inhibitor Cocktail, Basel, Switzerland, Cat. Nr.11836170001, Roche), using a small plastic homogenizer to mechanically disperse the tissue, followed by brief bursts of sonication on ice. The homogenate was spun at 15,000× *g* for 10 min at 4 °C to remove cell debris, and the supernatant was transferred to fresh tubes. A modified Bradford assay [99] was performed to determine protein concentration, and 50 μg of sample was removed for digestion. The samples were first reduced in the presence of 10 mM-DTT for 1 h at 30 °C, followed by reduction in the presence of 30 mM-iodoacetamide for 30 min in the dark. The samples were then diluted with 50 mM-TEAB to ensure that the urea concentration was 2M before setting up an overnight digestion at 37 °C with trypsin (Promega Sequencing grade, Cat. Nr. V5111) at a ratio of enzyme: protein of 1:50. The reaction was stopped the following morning by the addition of 2 uL trifluroacetic acid (TFA) to bring the pH to below pH 4. Digests were dried and resuspended in 0.5% TFA for de-salting with C18 ZipTips^TM^ (Milipore, Burlington, MA, USA, Cat. Nr.ZTC18S96). The desalted digests were subsequently re-suspended in 0.1% formic acid for loading onto individual EvoTips. Sample loading per tip was 480 ng for the hippocampus and 580 ng for the cortex.

### 4.9. Mass Spectrometry and nLC

The tryptic digests were run on a timsTOF Pro mass spectrometer (Bruker Daltonics, Bremen, Germany) coupled to the EvoSep One system (EvoSep BioSystems, Odense, Denmark). The peptides were separated on a reversed-phase C18 Endurance column (15 cm × 150 μM ID, C18, 1.9 μM) using the pre-set extended method. Mobile phases were 0.1% (*v*/*v*) formic acid in water (phase A) and 0.1% (*v*/*v*) formic acid in acetonitrile (phase B). The peptides were separated by an increasing gradient of mobile phase B for 88 min using a flow rate of 0.5 uL/min. The timsTOF Pro mass spectrometer was operated in positive ion polarity with TIMS (trapped ion mobility spectrometry) and PASEF (parallel accumulation serial fragmentation) modes enabled. The accumulation and ramp times for TIMS were both set to 100 ms, with an ion mobility (1/k0) range of 0.6 to 1.6 Vs/cm. Spectra were recorded in the mass range of 100 to 1700 m/z. The precursor (MS) intensity threshold was set to 1000, and the precursor target intensity was set to 20,000. Each PASEF cycle consisted of one MS ramp for precursor detection, followed by 5 PASEF MS/MS ramps, with a total cycle time of 1.03 s. Due to the size of the raw mass spec files, the data reduction option was selected before acquisition of the hippocampus samples, thereby reducing the file size from approximately 6 Gb to just over 2 Gb. Technical replicates were run on each sample.

### 4.10. Data Analysis

All data were searched with MaxQuant (version 2.0.3.0) against a Uniprot mouse reference proteome to which the human APP1 and PSEN genes were appended. Standard search settings were used, including allowing up to two missed cleavages for trypsin, variable modifications of oxidation on methionine and acetylation on the N terminus, and a fixed modification of carbamidomethylation on cysteine. The MBR (match between runs) and LFQ (label-free quantitation) options were also selected. The proteinGroups.txt output file generated by MaxQuant was used as input into Perseus (2.0 10.0). Data were filtered to remove any hits from the contaminants and the reversed sequence database. The samples were grouped into appropriate categories, the LFQ intensity values were log2 transformed, and proteins that did not appear in at least 70% of the samples were removed from the analysis. Any missing values that persisted after this filtering were imputed from a normal distribution. Data were normalised by subtraction of the median from column data.

### 4.11. Functional Enrichment Analysis

Ingenuity Pathway Analysis software 24.0.2 (Qiagen, Hilden, Germany) was used to identify networks and significantly enriched canonical pathways in cortical samples based on significantly (*p* < 0.05) differentially expressed proteins, based on two-tailed unpaired Student t-tests. Ingenuity Pathway Analysis software and the Ingenuity knowledge base (repository of data based on extensive information from published literature) was used for the prediction of associated canonical pathways and enriched networks. Analysis of the canonical pathways provided a *p*-value of overlap and a ratio indicating the strength of the association (this indicated the number of genes from the data set that map to the pathway divided by the total number of genes that map to the canonical pathway). Pathways with low *p*-values and high ratios may be the most significant candidates associated with the phenotype observed. Pathways with low *p*-values and scores of |Z| > 2 were prioritised, but those with scores of |Z| > 1.5 were also noted. The Ingenuity software generates an enrichment score for each network, which takes into account the number of eligible molecules/proteins in the network, its size, and the total number of eligible molecules analysed, as well as the total number of molecules in the Ingenuity knowledge base that could be included in networks.

Supplementary methods on mouse genotyping, breeding (Appendix A.1 and Appendix A.2) and preparation of amyloid derived diffusible ligands (Appendix A.3) can be found in Appendix A.

Statistical analysis: All graphs were plotted using GraphPad Prism 5.0 software (GraphPad Software Inc., Boston, MA, USA), and statistical analysis was also carried out using this software. The distribution of all datasets was assessed by the Shapiro–Wilk test and found to be normally distributed. Data in graphs are expressed as mean values ± standard error of the mean (SEM) of the replications for each experiment, represented as error bars unless otherwise stated. One-way ANOVA with Bonferroni post hoc test and two-tailed unpaired t-tests were used, with significance set at the 95% confidence interval.

## Data Availability

The data presented in this study are available on request from the corresponding author with the permission of Jazz Pharmaceuticals.

## Supplementary Materials

The following supporting information can be downloaded at: https://www.mdpi.com/article/10.3390/ijms26104944/s1. This includes information on alterations in mitochondrial proteins [100] caused by CBDA. In addition proteins involved in protein sorting, synaptogenesis and integrin signalling.

## Appendix A

Supplementary Methods

Mouse breeding and genotyping: Wild-type C57BL/6J and transgenic APPswe/PS1dE9 mice on a C57BL/6J background were used during this study. All animals were housed in the biomedical facility at University College Dublin (UCD), at 21 ± 1 °C, with a dark/light cycle of 12 h, and fed with chow and water ad libitum. Wild-type C57BL/6J mice purchased from the biomedical facility stock or from Charles River UK Limited were used for acute experiments with CBDA.

### Appendix A.1. APPswe/PSN1dE9 (APP/PS1)

Four heterozygous double-transgenic APPswe/PSN1dE9 (APP/PS1) male mice were purchased from The Jackson Laboratory and crossed with C57BL/6J females from Charles River UK Limited in the biomedical facility. The progeny of APP/PS1 mice bred on the C57BL/6J background were used to generate our transgenic colony. These mice have two transgenes inserted at a single locus. First, the humanised mouse mutant transgene Mo/HuAPP695swe, or the APP Swedish mutation of amyloid precursor protein, allows the mice to secrete human Aβ peptide, increasing the total amount of Aβ produced. Secondly, the PS1 sequence with deleted presenilin 1 (one of the four core proteins in the γ-secretase complex) in exon 9 increases the relative amount of Aβ_42_ produced compared to Aβ_40_. Male and female mice were separated after birth in groups of up to 5 mice per cage. All animals were identified with ear punches, and this tissue was later used for genotyping. In a cage, mice could display ear punches as follows: 1R (1 punch in the right ear), 1L (1 punch in the left ear), 1B (1 punch in both ears), 2R (2 punches in the right ear), or 2L (2 punches in the left ear). Regardless of the genotype, mice were kept together in their original cages and aged for 8 months before commencing chronic treatment with CBDA or vehicle.

Due to the fact that electrophysiology would be performed on a daily basis using tissue from one mouse, the breeding program also had to be staggered in order to provide a constant supply of animals that would be the correct age at the correct time. In addition, we expected that 25% of the mice carrying the transgene would die from spontaneous seizures during the first 8 weeks of life, which was a fact we also had to factor into the breeding program. Mice caged together received the same treatment. Mice were injected each morning, Monday to Friday, for 5 weeks. Injections were performed on a rolling basis, starting with one mouse each day, and euthanised two days after their last injection. The animals were anesthetised in an anaesthetic chamber under oxygen and isoflurane prior to decapitation using a guillotine. During the dissection, one hemibrain was used to prepare hippocampal slices for electrophysiology, and the other one was flash frozen in liquid nitrogen and stored at −80° for biochemical analyses.

Genotyping procedure: DNA extraction

DNA was extracted from ear-punch samples taken by the biomedical facility staff from all mice in the APP/PS1 colony. The Accustart II Mouse Genotyping Kit was used to genotype the samples. Briefly, tissue was first cleaned with 75% ethanol, followed by a wash with 1x phosphate-buffered saline (PBS) and elimination of excess of liquid. Once dried, the tissue was immersed in extraction reagent and incubated at 95 °C for 2 h until fully lysed. Finally, stabilisation buffer was added prior to storage at −20 °C.

Polymerase chain reaction (PCR): Two PCRs were performed on each DNA sample: one to amplify and determine the presence (or absence) of the human APPswe/PS1dE9 transgene, and another to amplify a control prion sequence present in all mice to ensure the presence of cDNA in the used sample.

### Appendix A.2. For Detection of Control DNA (324 bb)

Polymerase chain reaction (PCR) was performed using a thermal cycler program (master cycler gradient Eppendorf) for 35 cycles (each cycle: 95 °C for 2 min, 95 °C for 30 sec, 59.6 °C for 1 min, 72 °C for 3 min, 72 °C for 10 min) and held at 4 °C prior to electrophoresis. PCR was performed using two primer sets, one designed to amplify mouse Prn-p gene control DNA fragments (MPID1: ATAATCAGTGGAACAAGCCCA and MPID-2: GCAAAGAGCAACTGGTCTACTGTA purchased from Operon), and the other set designed to amplify the human APP695/PSN1dE9 cDNA (PrP-SJ=CCAAGCCTAGACCACGAGAATGC and S-36=CCGAGATCTCTGAAGTGAAGATGGATG purchased from MWG). Two separate sets of 25 μL PCR samples were prepared (first, 12.5 µL of GoTaq Green master mix (Promega, Southampton, UK), 10 pmol of MPID1 and MPID2, and 8.5 µL of DNA; second, 12.5 µL of TaqMix, 10 pmol of S-36 and PrP-SJ primers, and 8.5 µL of DNA extract) and run to avoid any cross-reactions between the PrP control and APP primer sets. Electrophoresis was performed using a 1.5% *w*/*v* agarose gel in 0.5 × Tris acetate–EDTA buffer (Sigma, UK) using a Helixx technologies iMy Run apparatus in 0.5× TAE buffer solution (Sigma, UK) at 100 V for 45 min. Ethidium bromide fluorescent bands were revealed using UV (λ = 260 nm) illumination using a Gene Genius Bioimaging system (Syngene; Cambridge UK). Photographs of each gel were stored using the GeneTrap acquisition program (Syngene; Cambridge UK).

### Appendix A.3. Preparation of Amyloid-Derived Diffusible Ligands (ADDLs) Aβ_1−42_

Synthetic human Aβ_1–42_ was synthesised and purified using reverse-phase HPLC by Dr. James Elliot at the ERI amyloid laboratory (Oxford, CT, USA). Aβ_1−42_ was dissolved in ice-cold HFIP (1,1,1,3,3,3-hexafluoro-2-propanol (Sigma-Aldrich, Gillingham, UK)), sonicated in an ultrasonic bath sonicator for 10 min, and incubated at room temperature for 1 h in glass HPLC tubes. The HFIP was then evaporated using a gentle stream of N_2_, and the remaining film was dissolved in anhydrous dimethylsulphoxide (DMSO) by vigorous vortexing. The solution was diluted in SILAC Advanced DMEM/F12 Flex Media (A2494301, Bio-Sciences), vortexed, incubated for 16 h at room temperature, snap frozen in liquid N_2_, and stored at −80 °C.
