# Supplementary material for: Cannabidiolic Acid Rescues Deficits in Hippocampal Long-Term Potentiation in Models of Alzheimer’s Disease: An Electrophysiological and Proteomic Analysis
Source: Int J Mol Sci. 2025 May 21;26(10):4944. doi: 10.3390/ijms26104944 (PMC12112199; doi:10.3390/ijms26104944)
Supplement: Supplementary file 1 [file ijms-26-04944-s001.zip › ijms-3592397-supplementary.pdf]

Supplementary Materials: Network enrichment analysis (Fig S1) shows significant alterations in the expression of mitochondrial proteins as key to the effect of CBDA in APP/PS1 mice (Cycs, Gstt1, mTOR, Ndufb6, Ndufs4, Pdhx, Pik3c3, Prkaa1, Prkaa2, Sdhc) with CBDA reversing the direction of expression change compared to that seen in APP/PS1 mice compared to wildtype controls. These mitochondrial proteins form part of processes such as oxidative phosphorylation, the constitution of Complexes I, II and IV of the electron transport chain. mTOR can regulate the translation of multiple electron-transport chain proteins, including Ndufb6 and Ndufs4, amongst others [96]. Fig S2. shows alterations in the expression of protein sorting proteins in the effect of CBDA in APP/PS1 mice with CBDA reversing the direction of expression change compared to that seen in APP/PS1 mice compared to wildtype controls. These proteins are associated with protein sorting in exocytic and endocytic pathways at the level of the Golgi apparatus complex, the plasma membrane and endosomes and are usually sorted into separate vesicle carriers. Fig S3. shows alterations in the expression of synaptogenesis associated proteins and the effect of CBDA in APP/PS1 mice. CBDA reverses the direction of expression change compared to that seen in APP/PS1(vehicle) mice compared to wildtype controls. These proteins form part of processes underlying the creation of new synapses. Fig S4. shows significant alterations in the expression of protein sorting proteins in the effect of CBDA in APP/PS1 mice with CBDA reversing the direction of expression change compared to that seen in APP/PS1 mice compared to wildtype controls. Integrin signalling proteins act both as adhesion and signalling elements and transduce mechano-transduction and biochemical signals across the plasma membrane. This requires both extracellular integrin domains and cytoskeletal protein elements which convey these signals.

Fig S 1 Mitochondrial Protein Expression

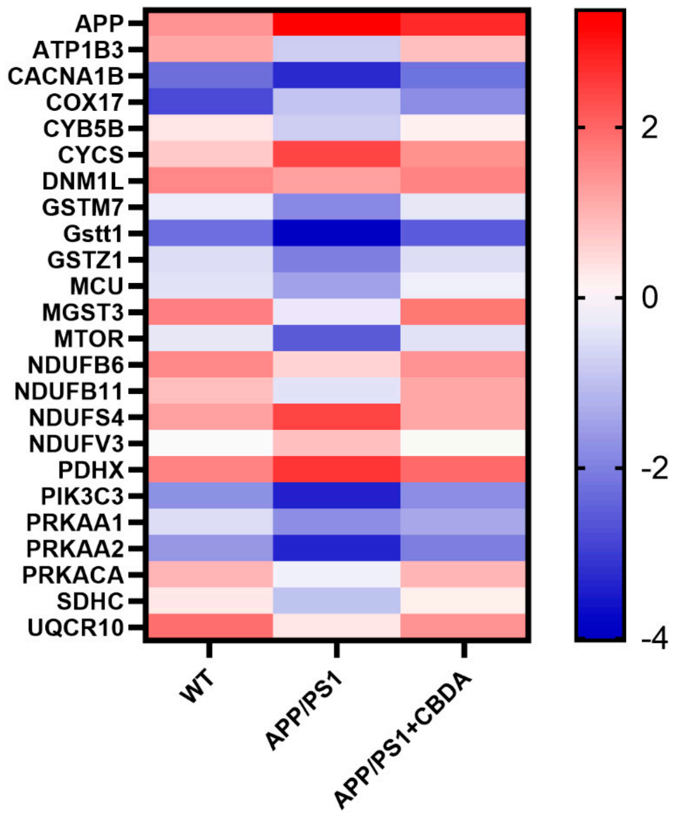

Figure S1. Heatmap plot of wildtype, APP/PS1 and CBDA-treated APP/PS1 mice on cortical mitochondrial protein expression levels compared to vehicle-treated wildtype mice. Data presented as Log_2 average intensity value. Proteins significantly differentially expressed in APP/PS1 compared to WT were APP, Cacna1b, Gstt1, mTOR, Ndufb6, Ndufs4, Pdhx, Pik3c3, Dnm1l, Prkaa1/2, and Sdhc. Comparing APP/PS1 to APP/PS1 CBDA Significantly differentially expressed proteins were Cacna1b, Dnm1l, GSTZ1, Pik3c3.Pdhx, mTOR, Ndufs4, Ndufs5, Sdhc.

Fig. S2. Protein Sorting and Processing

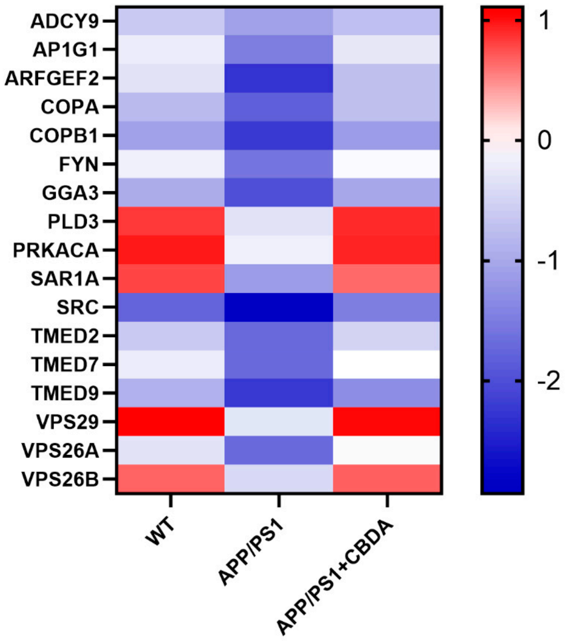

Figure S2. Heatmap plot of differential cortical protein expression of proteins associated with protein sorting / processing in vehicle-treated wildtype, vehicle-treated APP/PS1 and CBDA-treated APP/PS1 mice. Data presented as average Log_2 intensity. Comparing APP/PS1 to Wt. significantly differentially expressed proteins included Ap1g1, Argef2, Copb1, Tmed2 and Tmed9. Comparing APP/PS1 to APP/PS1 treated with CBDA, significantly differentially expressed proteins were Copb1, Ap1g1, Arfgef2, Src, Tmed2 and Tmed7.

Fig S3. Synaptogenesis Protein Expression

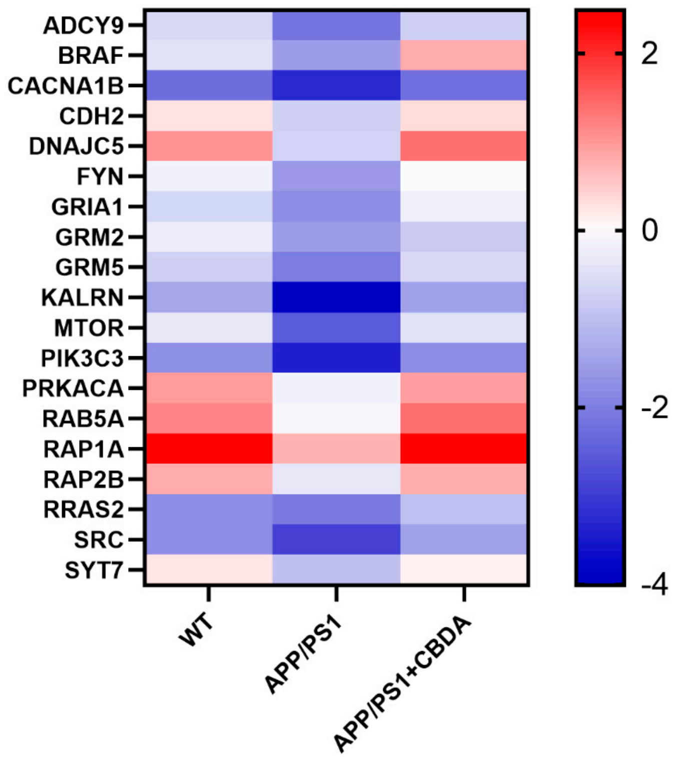

Figure S3. Heatmap plot of wildtype, APP/PS1 and CBDA-treated APP/PS1 mice on cortical synaptogenesis protein expression levels compared to vehicle-treated wildtype mice. Data presented as $\log_2$ average intensity value. Comparing APP/PS1 and WTt., significantly differentially expressed proteins were Gria1, Grm2, Grm5, Kalrn, mTOR, Pik3c3, Rras2, Src and Syt7. Comparing APP/PS1 to APP/PS1 CBDA treated significantly differentially expressed proteins were Gria1, Grm5, Kalrn, mTOR, Pik3c3, Src and Syt7.

Fig.S4 Integrin Signalling

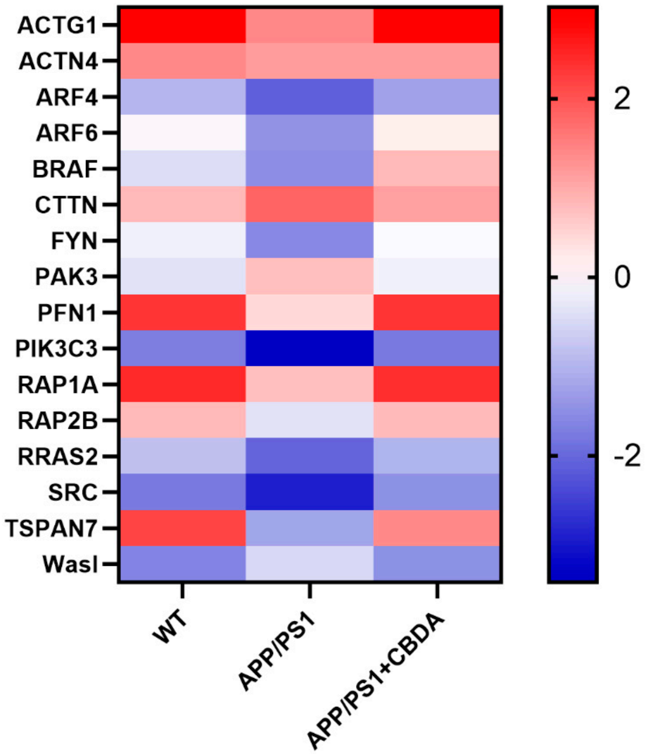

Figure S4. Heatmap plot of differential cortical protein expression of proteins associated with integrin signalling in vehicle-treated wildtype, vehicle-treated APP/PS1 and CBDA-treated APP/PS1 mice. Data presented as average log2 intensity value from Perseus. Comparing APP/PS1 to WT significantly differentially expressed proteins were Actn4, Cttn, Pik3c3, Pak3, Src and Tspan7. CBDA treatment of APP/PS1 changed the significant differential expression of Pak3, Pik3c3 and Src.

Table S1. proteins: Differentially expressed cortical proteins (students t-test in Perseus) comparing APP/PS1 (vehicle) to WT (vehicle) ranked by probability (-log10 p-value).

	APP/PS1 vs Wildtype			
	p-value	difference	gene name	Protein name
1	4.979	0.87	Atxn2	Ataxin-2
2	4.401	1.16	Pak3	Serine/threonine-protein kinase PAK 3
3	4.338	1.05	Hmgb1	High mobility group protein B1
4	4.276	0.58	Oat	Ornithine aminotransferase, mitochondrial
5	3.999	-1.25	Arhgap39	Rho GTPase-activating protein 39
6	3.703	0.89	Tppp3	Tubulin polymerization-promoting protein family member 3
7	3.702	1.45	Hnrnph2	Heterogeneous nuclear ribonucleoprotein H2
8	3.682	0.71	Snx3	Sorting nexin-3
9	3.617	0.95	Hspd1	60 kDa heat shock protein, mitochondrial
10	3.528	1.96	App	Amyloid Precursor Protein
11	3.514	-1.23	Rpl18	60S ribosomal protein L18
12	3.492	1.06	Ddah2	N(G),N(G)-dimethylarginine dimethylaminohydrolase 2
13	3.490	0.85	Eif3i	Eukaryotic translation initiation factor 3 subunit I
14	3.447	0.89	Sgtb	Small glutamine-rich tetratricopeptide repeat-containing protein beta
15	3.444	0.82	Cndp2	Cytosolic non-specific dipeptidase
16	3.436	1.19	Luc7l2	Putative RNA-binding protein Luc7-like 2
17	3.423	-0.78	Ube3a	Ubiquitin-protein ligase E3A
18	3.413	-0.64	Rap2a	Ras-related protein Rap-2a
19	3.408	0.93	Sfpq	Splicing factor, proline- and glutamine-rich
20	3.298	-1.33	Eif3l	Eukaryotic translation initiation factor 3 subunit L
21	3.285	1.07	Nucks1	Nuclear ubiquitous caseincycli/n-dependent kinase substrate 1
22	3.271	0.32	Napb	Beta-soluble NSF attachment protein
23	3.254	0.47	Uqcrc1	Cytochrome b-c1 complex subunit 1, mitochondrial
24	3.249	-1.03	Lancl2	LanC-like protein 2
25	3.146	-1.46	Fam49a	Protein FAM49A
26	3.118	0.92	Bckdha	2-oxoisovalerate dehydrogenase subunit alpha, mitochondrial
27	3.109	0.64	Metap2	Methionine aminopeptidase 2
28	3.099	1.86	Clu	Clusterin;Clusterin beta chain;Clusterin alpha chain
29	3.082	1.62	Gfap	Glial fibrillary acidic protein
30	3.064	1.00	Sh3bgrl3	SH3 domain-binding glutamic acid-rich-like protein 3
31	3.055	-1.54	Iars	Isoleucine--tRNA ligase, cytoplasmic
32	3.049	1.05	Cst3	Cystatin-C

33	3.034	-0.78	Dpp10	Inactive dipeptidyl peptidase 10
34	3.027	0.91	H2Ke6;Hsd17b8	Estradiol 17-beta-dehydrogenase 8
35	3.017	-2.01	Acsl4	Long-chain-fatty-acid--CoA ligase 4
36	3.014	-0.55	Slc6a1	Sodium- and chloride-dependent GABA transporter 1
37	2.998	-2.73	Pgrmc2	Membrane-associated progesterone receptor component 2
38	2.986	0.29	Akr1b1;Akr1b3	Aldose reductase
39	2.979	-1.99	Arfgef2	Brefeldin A-inhibited guanine nucleotide-exchange protein 2
40	2.951	-1.84	Gabbr2	Gamma-aminobutyric acid type B receptor subunit 2
41	2.943	-1.46	Mpc2	Mitochondrial pyruvate carrier 2
42	2.937	0.73	Fkbp4	Peptidyl-prolyl cis-trans isomerase FKBP4
43	2.936	0.59	Lmnb1	Lamin-B1
44	2.923	0.50	Hsp90b1	Endoplasmin
45	2.913	0.45	Pcmt1	Protein-L-isoaspartate O-methyltransferase
46	2.869	0.25	Gpr158	Probable G-protein coupled receptor 158
47	2.858	-1.24	Cadps2	Calcium-dependent secretion activator 2
48	2.812	-0.55	Edc4	Enhancer of mRNA-decapping protein 4
49	2.809	-0.97	Cacng3	Voltage-dependent calcium channel gamma-3 subunit
50	2.801	0.99	Timm10b	Mitochondrial import inner membrane subunit Tim10 B
51	2.800	0.95	Hagh	Hydroxyacylglutathione hydrolase
52	2.783	0.83	Rps19	40S ribosomal protein S19
53	2.725	0.71	Sh3glb2	EndophilinB2
54	2.724	1.16	Tppp	Tubulin polymerization-promoting protein
55	2.723	-1.60	Bre	BRCA1-A complex subunit BRE
56	2.721	1.18	Pfdn2	Prefoldin subunit 2
57	2.707	1.13	Nedd8	NEDD8
58	2.694	-0.20	Otub1	Ubiquitin thioesterase OTUB1
59	2.689	-0.99	Kbp	KIF1-binding protein
60	2.688	-0.60	Fam213a	Redox-regulatory protein FAM213A
61	2.679	-1.14	Gria1	AMPA receptor subunit1
62	2.678	1.17	Nudt9	ADP-ribose pyrophosphatase, mitochondrial
63	2.664	0.58	Srp72	Signal recognition particle subunit SRP72
64	2.657	0.95	Srsf3	Serine/arginine-rich splicing factor 3
65	2.653	-1.19	Pdk1	Pyruvate dehydrogenase kinase isozyme 1, mitochondrial
66	2.653	0.92	Atxn2l	Ataxin-2-like protein
67	2.647	-1.20	Agk	Acylglycerol kinase, mitochondrial
68	2.634	0.86	Eps15l1	Epidermal growth factor receptor substrate 15-like 1
69	2.629	0.95	Ak1	Adenylate kinase isoenzyme 1
70	2.628	0.91	Tpd52	Tumour protein D52

71	2.618	0.71	Cmpk1	UMP-CMP kinase
72	2.599	1.05	Pfdn6	Prefoldin subunit 6
73	2.588	0.78	Psmc4	26S protease regulatory subunit 6B
74	2.576	-0.76	Ap2a2	AP-2 complex subunit alpha-2
75	2.573	1.02	Serpina1a	Alpha-1-antitrypsin 1-3
76	2.545	0.58	Hdlbp	Vigilin
77	2.538	-3.19	Clptm1	Cleft lip and palate transmembrane protein 1 homolog
78	2.538	0.96	Strap	Serine-threonine kinase receptor-associated protein
79	2.533	-1.52	Eif3m	Eukaryotic translation initiation factor 3 subunit M
80	2.527	-0.57	Vdac3	Voltage-dependent anion-selective channel protein 3
81	2.518	-0.51	Actn1	Actinin alpha 1
82	2.515	0.94	Slc9a3r1	Na(+)/H(+) exchange regulatory cofactor NHE-RF1
83	2.500	-0.78	Pde1a	Calcium/calmodulin-dependent 3,5-cyclic nucleotide 1A
84	2.474	-3.37	Rps16	40S ribosomal protein S16
85	2.470	-1.24	Tm9sf2	Transmembrane 9 superfamily member 2
86	2.454	1.85	Dkk3	Dickkopf-related protein 3
87	2.447	1.65	Pfdn1	Prefoldin subunit 1
88	2.442	-1.39	Tnpo2	Transportin2
89	2.441	0.86	Txndc5	Thioredoxin domain-containing protein 5
90	2.424	1.17	Tpm4	Tropomyosin alpha-4 chain
91	2.414	-1.76	Aldh3a2	Fatty aldehyde dehydrogenase
92	2.404	1.18	Tbcb	Tubulin-folding cofactor B
93	2.393	0.77	Hnrnpa0	Heterogeneous nuclear ribonucleoprotein A0
94	2.388	0.98	Lmnb2	Lamin-B2
95	2.384	0.71	Napg	Gamma-soluble NSF attachment protein
96	2.379	0.58	Slk	STE20-like serine/threonine-protein kinase
97	2.378	0.87	Aimp1	Aminoacyl tRNA synthase complex-interacting protein 1
98	2.376	1.20	Ndufs4	NADH dehydrogenase iron-sulfur protein 4, mitochondrial
99	2.375	-1.62	Faah	Fatty-acid amide hydrolase 1
100	2.365	-1.31	Slc25a12	Calcium-binding mitochondrial carrier protein Aralar1

Table S2. Top Pathways identified using ingenuity IPA analysis comparing APP/PS1 (vehicle) vs. WT (vehicle) (Cortical tissue).

Pathway	p value	Z-score	Ratio
Mitochondrial Dysfunction	10.2	1.4	0.0727
Protein Sorting Signaling Pathway	8.92	-4.12	0.0955
Remodeling of Epithelial Adherens Junctions	8.39	N/A	0.162
Post-translational protein phosphorylation	7.25	2.31	0.112
Integrin Signaling	6.98	-1.29	0.0755
Synaptogenesis Signaling Pathway	6.63	-4.12	0.0603
Regulation of Insulin-like Growth Factor (IGF)	6.53	2.31	0.0968
Endocannabinoid Developing Neuron Pathway	6.42	-2.71	0.0945
mTOR Signaling	6.17	-1.00	0.0701
EIF2 Signaling	5.78	-2.83	0.0652
Macropinocytosis Signaling	5.78	-2.65	0.118
Protein folding	5.75	1.26	0.102
Eukaryotic Translation Initiation	5.73	-2.11	0.0902
Circadian Rhythm Signaling	5.62	N/A	0.0597
Estrogen Receptor Signaling	5.53	-3.87	0.0489
Electron transport, ATP synthesis	5.53	-0.90	0.0859
TBC/RABGAPs	5.49	-2.65	0.159
Opioid Signaling Pathway	5.38	-2.84	0.0571
Oxidative Phosphorylation	5.22	-0.33	0.0893
GABAergic Receptor Signaling Pathway (Enhanced)	5.18	-1.51	0.0791
Serotonin Receptor Signaling	5.18	-3.71	0.0448
Gαi Signaling	5.15	-1.00	0.0786
HER-2 Signaling in Breast Cancer	5.15	-2.50	0.0617
Apelin Endothelial Signaling Pathway	5.12	-2.53	0.078
Germ Cell-Sertoli Cell Junction Signaling	5.08	N/A	0.0706
Intra-Golgi and retrograde Golgi-to-ER traffic	5	-3.05	0.064
Sertoli Cell-Germ Cell Junction Signaling Pathway (Enha	4.96	-0.53	0.0593

Table S3. 100 Differentially expressed cortical proteins (students t-test in Perseus) ranked by p-value (- $\log_{10}$ p-value) comparing APP/PS1 CBDA vs APP/PS1 vehicle.

	p-value	Difference	Gene names	Protein names
1	6.346	-1.00	Hmgb1	High mobility group protein B1
2	3.642	-0.87	Rps19	40S ribosomal protein S19
3	3.463	0.49	Dpp6	Dipeptidyl aminopeptidase-like protein 6
4	3.421	0.60	Camk2d	Calcium/calmodulin-dependent protein kinase type II delta
5	3.270	-0.69	Eif3i	Eukaryotic translation initiation factor 3 subunit I
6	3.079	0.91	Lancl2	LanC-like protein 2
7	3.055	1.42	Fam49a	Protein FAM49A
8	3.053	-0.75	Sgtb	Small glutamine-rich tetratricopeptide repeat- beta
9	3.011	2.00	Acsl4	Long-chain-fatty-acid--CoA ligase 4
10	2.973	1.46	Iars	Isoleucine--tRNA ligase, cytoplasmic
11	2.946	0.80	Ube3a	Ubiquitin-protein ligase E3A
12	2.913	1.21	Agk	Acylglycerol kinase, mitochondrial
13	2.893	1.57	Gria1	AMPA receptor subunit 1
14	2.863	-0.85	Sfpq	Splicing factor, proline- and glutamine-rich
15	2.859	-0.89	Atxn2	Ataxin-2
16	2.854	1.42	Tm9sf2	Transmembrane 9 superfamily member 2
17	2.850	-0.81	Timm10b	Mitochondrial import inner membrane translocase subunit Tim10 B
18	2.823	-0.72	Sh3glb2	Endophilin-B2
19	2.809	2.61	Pgrmc2	Membrane-associated progesterone receptor component 2
20	2.806	0.53	Actr2	Actin-related protein 2
21	2.777	-0.89	Mpp1	55 kDa erythrocyte membrane protein
22	2.755	1.21	Tmed2	Transmembrane emp24 domain-containing protein 2
23	2.744	-0.62	Hist1h4a	Histone H4
24	2.743	1.59	Bre	BRCA1-A complex subunit BRE
25	2.739	0.76	Fam213a	Redox-regulatory protein FAM213A
26	2.699	-0.67	Ddah2	N(G),N(G)-dimethylarginine dimethylaminohydrolase 2
27	2.665	-0.50	Myef2	Myelin expression factor 2
28	2.630	0.65	Acss1	Acetyl-coenzyme A synthetase 2-like, mitochondrial
29	2.621	-0.20	Sdha	Succinate dehydrogenase flavoprotein subunit

30	2.603	-1.35	Stmn1	Stathmin
31	2.599	0.76	Ap2a2	AP-2 complex subunit alpha-2
32	2.591	-0.72	Gas7	Growth arrest-specific protein 7
33	2.582	-0.91	Lmnb2	Lamin-B2
34	2.522	-0.51	Rps27a	Ubiquitin-40S ribosomal protein S27a
35	2.506	0.75	Rab12	Ras-related protein Rab-12
36	2.503	1.65	Eif3m	Eukaryotic translation initiation factor 3 subunit M
37	2.492	-0.59	Cndp2	Cytosolic non-specific dipeptidase
38	2.470	-0.82	Hspd1	60 kDa heat shock protein, mitochondrial
39	2.466	-0.77	Hagh	Hydroxyacylglutathione hydrolase, mitochondrial
40	2.462	-0.84	Luc7l2	Putative RNA-binding protein Luc7-like 2
41	2.451	0.34	Sdr39u1	Epimerase family protein SDR39U1
42	2.424	-0.65	Atxn2l	Ataxin-2-like protein
43	2.408	-0.90	Lmna	Prelamin-A/C;Lamin-A/C
44	2.404	-0.29	Usp5	Ubiquitin carboxyl-terminal hydrolase
45	2.404	0.73	Abcf2	ATP-binding cassette sub-family F member 2
46	2.402	-0.73	Psmc4	26S protease regulatory subunit 6B
47	2.394	-1.29	Ndufs4	NADH dehydrogenaseon-sulfur protein 4, mitochondrial
48	2.393	0.61	Pde2a	cGMP-dependent 3,5-cyclic phosphodiesterase
49	2.369	0.75	Asl	Argininosuccinate lyase
50	2.362	-0.87	Ak1	Adenylate kinase isoenzyme 1
51	2.352	1.38	Mtmr2	Myotubularin-related protein 2
52	2.348	-0.55	Caskin1	Caskin-1
53	2.340	-1.35	Tpm1	Tropomyosin 1
54	2.320	-0.72	Cmpk1	UMP-CMP kinase
55	2.314	1.59	Arfgef2	Brefeldin A-inhibited guanine nucleotide-exchange protein 2
56	2.312	-1.08	Nefm	Neurofilament medium polypeptide
57	2.312	-0.83	Strap	Serine-threonine kinase receptor-associated protein
58	2.309	-0.75	Csrp1	Cysteine and glycine-rich protein 1
59	2.309	-0.78	Eps15l1	Epidermal growth factor receptor substrate 15-like 1
60	2.305	-0.92	Pak3	Serine/threonine-protein kinase PAK 3
61	2.305	0.40	Ndufa12	NADH dehydrogenase 1 alpha subcomplex subunit 12

62	2.305	-0.48	Srrt	Serrate RNA effector molecule homolog
63	2.282	-0.64	Dtd1	D-tyrosyl-tRNA(Tyr) deacylase 1
64	2.267	-0.66	Tbcb	Tubulin-folding cofactor B
65	2.263	1.55	Pdpr	Pyruvate dehydrogenase phosphatase regulatory subunit
66	2.254	-1.03	Eif4b	Eukaryotic translation initiation factor 4B
67	2.239	-0.86	Slc9a3r1	Na(+)/H(+) exchange regulatory cofactor NHE-RF1
68	2.236	-0.55	Sf3a1	Splicing factor 3A subunit 1
69	2.233	-0.76	Metap2	Methionine aminopeptidase 2
70	2.226	-0.54	Slk	STE20-like serine/threonine-protein kinase
71	2.224	-0.76	Rbbp4	Histone-binding protein RBBP4
72	2.221	1.01	Clpb	Caseinolytic peptidase B protein homolog
73	2.211	-0.73	Eno2	Gamma-enolase;Enolase
74	2.209	0.86	Ap1m1	AP-1 complex subunit mu-1
75	2.204	-0.78	Mdh2	Malate dehydrogenase, mitochondrial
76	2.188	1.39	Faah	Fatty-acid amide hydrolase 1
77	2.187	2.79	Clptm1	Cleft lip and palate transmembrane protein 1 homolog
78	2.179	0.62	Apba2	Amyloid beta A4 precursor protein-binding family A member 2
79	2.177	1.35	Slc25a25	Calcium-binding mitochondrial carrier protein SCaMC-2
80	2.176	0.73	Pde1a	Calcium/calmodulin-dependent 3,5-cyclic phosphodiesterase
81	2.173	-1.03	Tpm4	Tropomyosin alpha-4 chain
82	2.173	0.57	Psmd2	26S proteasome non-ATPase regulatory subunit 2
83	2.166	-0.46	Plec	Plectin
84	2.152	1.27	Cadps2	Calcium-dependent secretion activator 2
85	2.145	0.55	Ctnna2	Catenin alpha-2
86	2.123	-0.74	Mtfr1l	Mitochondrial fission regulator 1-like
87	2.118	-0.54	Lmnb1	Lamin-B1
88	2.117	-0.54	Snx3	Sorting nexin-3
89	2.112	-0.99	Hnrnph2	Heterogeneous nuclear ribonucleoprotein H2
90	2.109	1.32	Slc9a6	Sodium/hydrogen exchanger
91	2.107	0.96	Stxbp5	Syntaxin-binding protein 5
92	2.103	0.86	Vps35	Vacuolar protein sorting-associated protein 35
93	2.102	-1.00	Nedd8	NEDD8

94	2.097	1.36	Srp68	Signal recognition particle subunit SRP68
95	2.096	0.47	Camkv	CaM kinase-like vesicle-associated protein
96	2.090	-0.90	Crip2	Cysteine-rich protein 2
97	2.084	-0.90	Sh3bgrl3	SH3 domain-binding glutamic acid-rich-like protein 3
98	2.083	0.93	Eif3l	Eukaryotic translation initiation factor 3 subunit L
99	2.080	-0.66	L1cam	Neural cell adhesion molecule L1
100	2.073	1.21	Ap1g1	AP-1 complex subunit gamma-1

Table S4. Top Pathways identified in cortical tissue using ingenuity IPA analysis comparing APP/PS1 CBDA treated vs. APP/PS1 vehicle treated groups.

Pathway	- log10 (p-value)	z score	ratio			
Remodeling of Epithelial Adherens Junctions	9.2	N/A	0.16			
Protein Sorting Signaling Pathway	9.16	4.00	0.09			
Synaptogenesis Signaling Pathway	8.62	3.77	0.06			
Integrin Signaling	8.06	1.29	0.08			
Macropinocytosis Signaling	7.52	2.12	0.13			
Circadian Rhythm Signaling	7.4	N/A	0.06			
Endocannabinoid Developing Neuron Pathway	7.26	2.11	0.09			
Paxillin Signaling	7.08	1.51	0.10			
Germ Cell-Sertoli Cell Junction Signaling	6.72	N/A	0.08			
Virus Entry via Endocytic Pathways	6.64	2.71	0.09			
Eicosanoid Signaling	6.39	3.50	0.06			
Antiproliferative Role of Somatostatin Receptor 2	6.37	2.12	0.12			
Activation of NMDA receptors and postsynaptic events	6.05	2.12	0.11			
Mitochondrial Dysfunction	5.87	-0.73	0.05			
Sertoli Cell-Germ Cell Junction Signaling Pathway (Enhan	5.85	0.53	0.06			
Signaling by ROBO receptors	5.77	1.26	0.09			
Sertoli Cell-Sertoli Cell Junction Signaling	5.7	2.14	0.06			
IL-8 Signaling	5.68	1.73	0.06			
VEGF Signaling	5.44	2.12	0.09			
HER-2 Signaling in Breast Cancer	5.31	1.73	0.06			
Serotonin Receptor Signaling	5.22	3.44	0.04			
Autism Signaling Pathway	5.17	2.32	0.05			
Signaling by VEGF	5.13	1.00	0.08			
NRF2-mediated Oxidative Stress Response	5.1	1.63	0.05			
GABAergic Receptor Signaling Pathway (Enhanced)	5.07	1.26	0.07			
Gαi Signaling	5.04	0.33	0.07			
Apelin Endothelial Signaling Pathway	5.01	1.67	0.07			

Table S5. 100 proteins ranked by -log10 p value comparing APP/PS1 CBDA treated to Wt vehicle treated cortex. (Only the top 4 proteins were significantly altered).

	p-value	Difference	Gene names	Protein names
1	3.767	1.314	App	Amyloid Precursor Protein
2	3.634	0.920	C1qc	Complement C1q subcomponent subunit C
3	3.160	1.324	Pcsk1n	ProSAAS
4	3.106	0.727	Chordc1	Cysteine and histidine-rich domain-containing protein 1
5	2.963	-0.361	Fibp	Acidic fibroblast g factor intracellular-binding protein
6	2.952	0.890	Apoe	Apolipoprotein E
7	2.869	1.552	Gfap	Glial fibrillary acidic protein
8	2.770	-0.358	Pdcd6	Programmed cell death protein 6
9	2.697	-0.262	Dynll2	Dynein light chain 2, cytoplasmic
10	2.695	0.823	Spock2	Testican-2
11	2.667	0.803	Grn	Granulins
12	2.630	0.375	Rpl23	60S ribosomal protein L23
13	2.585	0.358	Cyb5r3	NADH-cytochrome b5 reductase 3
14	2.555	-0.297	Cdk5	Cyclin-dependent-like kinase 5
15	2.452	0.351	Luc7l2	Putative RNA-binding protein Luc7-like 2
16	2.433	0.479	Sdr39u1	Epimerase family protein SDR39U1
17	2.393	-0.596	Dlgap2	Disks large-associated protein 2
18	2.382	0.339	Camkv	CaM kinase-like vesicle-associated protein
19	2.365	0.289	Idh3g	Isocitrate dehydrogenase [NAD] subunit gamma 1
20	2.346	-0.232	Myo5a	Unconventional myosin-Va
21	2.308	-0.352	Rhot1	Mitochondrial Rho GTPase 1
22	2.294	-0.288	Hmox2	Heme oxygenase 2
23	2.260	0.521	Ube2l3	Ubiquitin-conjugating enzyme E2 L3
24	2.232	0.198	Cep170	Centrosomal protein of 170 kDa
25	2.152	-0.282	Gnb5	Guanine nucleotide-binding protein subunit beta-5
26	2.140	0.561	Gpc1	Glypican-1;Secreted glypican-1
27	2.077	0.350	Slc8a1	Sodium/calcium exchanger 1
28	2.049	0.394	Ddah2	N(G),N(G)-dimethylarginine dimethylaminohydrolase 2
29	2.049	-0.299	Gucy1a2	Guanylate cyclase 1 soluble subunit
30	2.042	0.467	Pafah1b3	Platelet-activating factor acetylhydrolase IB gamma

31	2.030	0.403	Pkm	Pyruvate kinase PKM
32	2.027	0.358	Flot2	Flotillin-2
33	2.020	0.449	Aimp1	Aminoacyl tRNA synthase complex
34	1.967	0.221	Kif21a	Kinesin-like protein KIF21A
35	1.956	0.420	Dpysl3	Dihydropyrimidinase-related protein 3
36	1.939	0.533	Cpe	Carboxypeptidase E
37	1.866	-0.290	Epn2	Epsin-2
38	1.863	0.655	Scp2	Non-specific lipid-transfer protein
39	1.853	0.496	Hyou1	Hypoxia up-regulated protein 1
40	1.849	0.566	Ubqln2	Ubiquilin-2
41	1.834	-0.413	Adam23	Disintegrin/ metalloproteinase domain protein 23
42	1.821	-0.483	Kctd16	BTB/POZ domain-containing protein KCTD16
43	1.799	0.636	Nptxr;Npcd	Neuronal pentraxin receptor
44	1.792	0.366	Haghl	Hydroxyacylglutathione hydrolase-like protein
45	1.785	-0.169	Gps1	COP9 signalosome complex subunit 1
46	1.780	0.398	Acot2	Acyl-coenzyme A thioesterase 2, mitochondrial
47	1.774	0.423	Rab3b	Ras-related protein Rab-3B
48	1.773	1.346	Psmf1	Proteasome inhibitor PI31 subunit
49	1.744	-0.424	Dgkh	Diacylglycerol kinase
50	1.744	1.104	Itm2c	Integral membrane protein 2C;CT-BRI3
51	1.734	-0.251	Rpl10a	Ribosomal protein;60S ribosomal protein L10a
52	1.727	0.730	Gcn1l1	Activator of the EIF2AK4/GCN
53	1.726	-0.768	Rgs6	Regulator of G-protein signaling 6
54	1.718	0.822	Clu	Clusterin;Clusterin beta chain;Clusterin alpha chain
55	1.714	-0.319	Kcnd2	Potassium voltage-gated channel subfamily D member 2
56	1.712	1.030	Dhrs4	Dehydrogenase/reductase SDR family member 4
57	1.705	-0.247	Mat2a	S-adenosylmethionine synthase isoform type-2
58	1.694	0.331	Acat2;Acat3	Acetyl-CoA acetyltransferase, cytosolic
59	1.691	-0.377	Camkk2	Calcium/calmodulin-dependent protein kinase kinase 2
60	1.670	-0.726	Cyth2	Cytohesin-2
61	1.664	-0.235	Rgs7	Regulator of G-protein signaling 7
62	1.660	-0.177	Nmt2	Glycylpeptide N-tetradecanoyltransferase

63	1.656	0.643	Calu	Calumenin
64	1.648	-0.303	Tuba4a	Tubulin alpha-4A chain
65	1.644	0.548	Serpina1a;Serpina1c	Alpha-1-antitrypsin 1-3;Alpha-1-antitrypsin 1-1
66	1.638	0.421	Prdx1	Peroxiredoxin-1
67	1.638	0.252	Glg1	Golgi apparatus protein 1
68	1.631	0.242	Serbp1	Plasminogen activator inhibitor 1 RNA-binding protein
69	1.623	0.294	Snx27	Sorting nexin-27
70	1.623	0.284	Txndc5	Thioredoxin domain-containing protein 5
71	1.614	-0.221	Csnk2a2	Casein kinase II subunit alpha
72	1.603	-0.349	Rheb	GTP-binding protein Rheb
73	1.591	0.477	Nln	Neurolysin, mitochondrial
74	1.590	0.299	Cttn	Src substrate cortactin
75	1.587	0.321	Ppa1	Inorganic pyrophosphatase
76	1.586	0.760	Chgb	Secretogranin-1;CCB peptide;PE-11
77	1.584	-0.289	Cops2	COP9 signalosome complex subunit 2
78	1.583	1.575	Tjp2	Tight junction protein ZO-2
79	1.554	-0.270	Dnm1	Dynamin-1
80	1.547	-0.180	Cadm3	Cell adhesion molecule 3
81	1.547	1.026	Cox17	Cytochrome c oxidase copper chaperone
82	1.547	-0.358	Igsf8	Immunoglobulin superfamily member 8
83	1.542	-0.451	Nptn	Neuroplastin
84	1.538	0.481	Appl2	DCC-interacting protein 13-beta
85	1.537	-0.389	Armc8	Armadillo repeat-containing protein 8
86	1.535	0.238	Mblac2	Metallo-beta-lactamase domain-containing protein 2
87	1.531	0.211	Hgs	Hepatocyte growth factor-regulated tyrosine kinase
88	1.526	-0.259	Ppp1cb	Serine/threonine-protein phosphatase PP1-beta
89	1.526	0.595	Atp9a	Phospholipid-transporting ATPase
90	1.525	0.262	Blvrb	Flavin reductase (NADPH)
91	1.523	-0.508	Fbxl16	F-box/LRR-repeat protein 16
92	1.522	-0.320	Slc25a1	Tricarboxylate transport protein, mitochondrial
93	1.522	-0.099	Pfkp	ATP-dependent 6-phosphofructokinase
94	1.518	-0.229	Slc25a12	Calcium-binding mitochondrial carrier protein Aralar1

95	1.515	0.455	Iqsec3	IQ motif and SEC7 domain-containing protein 3
96	1.510	-0.266	Rph3a	Rabphilin-3A
97	1.507	-0.280	Brsk2	Serine/threonine-protein kinase BRSK2
98	1.500	-0.267	Tecpr1	Tectonin beta-propeller repeat-containing protein 1
99	1.500	-0.347	Pdp1	Pyruvate dehydrogenase-phosphatase 1
100	1.486	-0.430	Cacnb4	Voltage-dependent L-type calcium channel beta-4

Table S6. Pathways identified using IPA ingenuity comparing APP/PS1 (CBDA treated) vs. WT. Control vehicle treated cortical tissue P-Value = -log10 (P-value). No z-score.

Pathway	-log10 p value	z score
Eukaryotic Translation Termination	4.52	
Eukaryotic Translation Elongation	4.5	
Response of EIF2AK4 (GCN2) to amino acid deficiency	4.4	
Selenoamino acid metabolism	4.35	
SRP-dependent cotranslational protein targeting to membrane	4.26	
Nonsense-Mediated Decay (NMD)	4.23	
Eukaryotic Translation Initiation	4.18	
Relaxin Signaling	3.87	
Major pathway of rRNA processing in the nucleolus and cytosol	3.64	
EIF2 Signaling	3.37	
cAMP-mediated signaling	3.33	
Endocannabinoid Developing Neuron Pathway	2.51	
Gαs Signaling	2.51	
TYSND1 cleaves peroxisomal proteins	2.34	
Cardiac β-adrenergic Signaling	2.21	
Endothelin-1 Signaling	2.15	
IL-8 Signaling	2.08	
Multiple Sclerosis Signaling Pathway	2.04	
G-Protein Coupled Receptor Signaling	1.99	
Colorectal Cancer Metastasis Signaling	1.87	
Peroxisomal lipid metabolism	1.84	

Eicosanoid Signaling	1.84
Opioid Signaling Pathway	1.84
Myelination Signaling Pathway	1.72
EGR2 and SOX10-mediated initiation of Schwann cell myelination	1.72
Transcriptional Regulation by E2F6	1.65
ERCC6 (CSB) and EHMT2 (G9a) positively regulate rRNA expression	1.62

Levels of soluble Aβ 40 measured using ELIZA across all treatment groups were similar. Vehicle treated APP/PS1 (17.46 ± 2.35 *n*=9); those treated with CBDA were similar, 1mg/kg CBDA (15.97± 2.01, *n*=9), 10mg/kg (16.9 1. ±86, *n*=10) and 30mg/kg (16.79 ± 2/1 *n*=10)
